# A Catalog of Proteins Expressed in the AG Secreted Fluid during the Mature Phase of the Chinese Mitten Crabs (*Eriocheir sinensis*)

**DOI:** 10.1371/journal.pone.0136266

**Published:** 2015-08-25

**Authors:** Lin He, Qing Li, Lihua Liu, Yuanli Wang, Jing Xie, Hongdan Yang, Qun Wang

**Affiliations:** School of Life Science, East China Normal University, Shanghai 200241, China; University of Nevada School of Medicine, UNITED STATES

## Abstract

The accessory gland (AG) is an important component of the male reproductive system of arthropods, its secretions enhance fertility, some AG proteins bind to the spermatozoa and affect its function and properties. Here we report the first comprehensive catalog of the AG secreted fluid during the mature phase of the Chinese mitten crab (*Eriocheir sinensis*). AG proteins were separated by one-dimensional gel electrophoresis and analyzed by reverse phase high-performance liquid chromatography coupled with tandem mass spectrometry (HPLC-MS/MS). Altogether, the mass spectra of 1173 peptides were detected (1067 without decoy and contaminants) which allowed for the identification of 486 different proteins annotated upon the NCBI database (http://www.ncbi.nlm.nih.gov/) and our transcritptome dataset. The mass spectrometry proteomics data have been deposited at the ProteomeXchange with identifier PXD000700. An extensive description of the AG proteome will help provide the basis for a better understanding of a number of reproductive mechanisms, including potentially spermatophore breakdown, dynamic functional and morphological changes in sperm cells and sperm acrosin enzyme vitality. Thus, the comprehensive catalog of proteins presented here can serve as a valuable reference for future studies of sperm maturation and regulatory mechanisms involved in crustacean reproduction.

## Introduction

Accessory glands in the male reproductive system secrets proteins or other molecules that can support sperm improve fertility. For example, in mammals, the epididymis has numerous inter-related functions, including reabsorption of the fluid secreted by the seminiferous tubules, as well as the maturation and storage of sperm. During epididymal transit, spermatozoa bind to proteins that were secreted by the epididymis via epididymosomal transport. These proteins can regulate the sperm motility or maturation [1]. The AG is responsible for the synthesis and secretion of a large number of seminal fluid proteins (SFPs). When transferred during mating, these molecules exert wide-ranging effects on female reproductive activities, and they improve the male's chances of siring a significant proportion of the female's offspring[2,3]. AG secretions can potentially affect virtually all aspects of the female's reproductive activity. In *Drosophila melanogaster*, transfer of SFPs from male to female during mating induces physiological changes in mated females, including increased egg production and reduced receptivity to remate [4].

**Fig 1:**
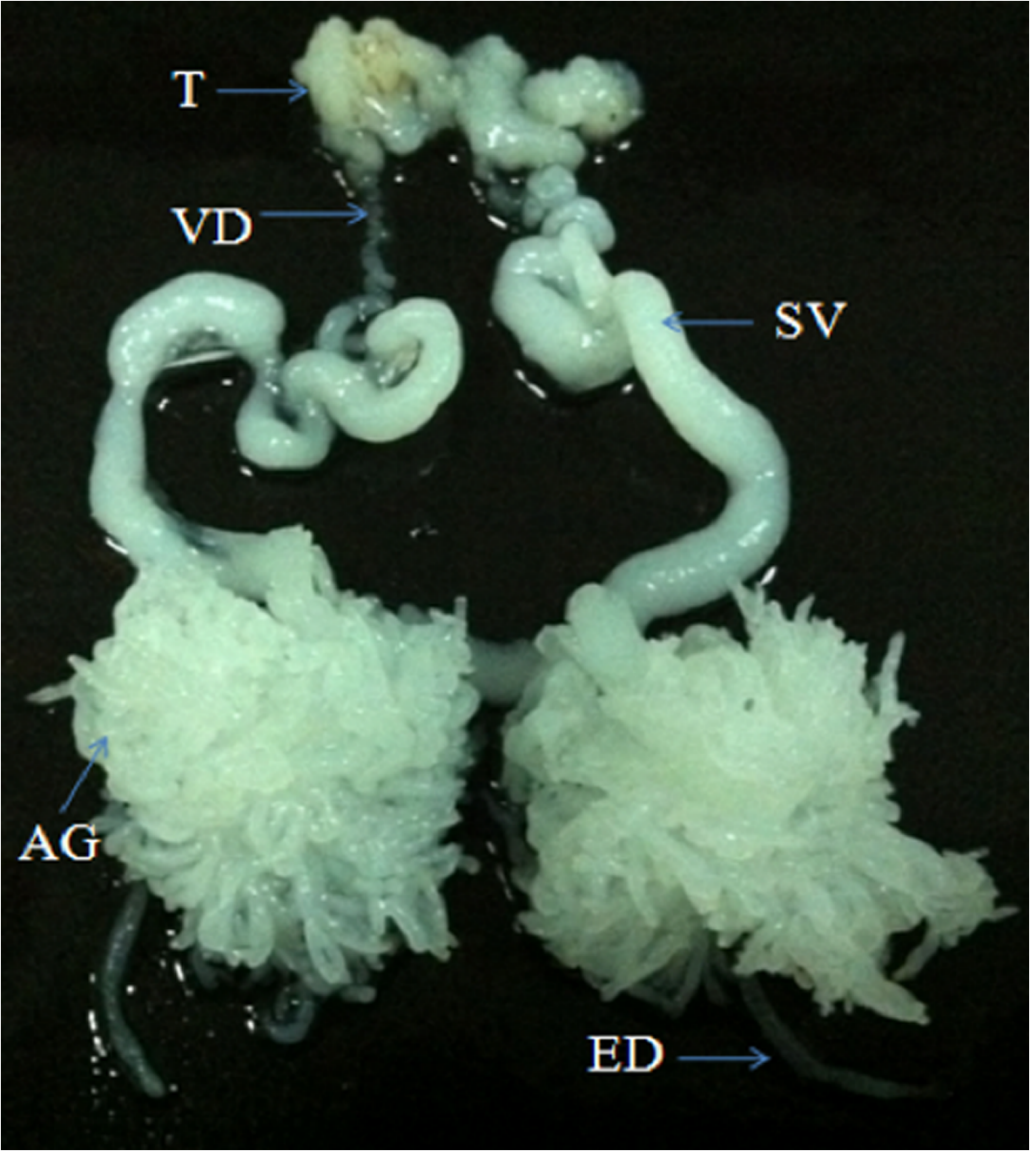
Photograph of the male reproductive system in the *E*. *sinensis*. The male reproductive system of this crab consists of a pair of testes (T), vas deferens (VD), seminal vesicles (SV), accessory glands (AG) and ejaculatory duct (ED).

*Eriocheir sinensis*, the Chinese mitten crab (Milne Edwards, 1853) is an economically important aquaculture species in China. Research on male reproductive biology of this crab mainly has focused on its spermatogenesis, spermatophore formation, spermatozoa ultrastructure, or spermatozoa metabolism. Transcriptome analysis of Chinese mitten crab testis [5] and AG [6] are newly available, opening up opportunities to learn more about these tissues’ functions. In the *E*. *sinensis* male, the reproductive system consists of a pair of testes with vas deferens, seminal vesicles, a pair of AG and a single ejaculatory duct (Fig 1). Mature male Chinese mitten crabs have a pair of well-developed AGs that open at the junction of seminal vesicles and the ejaculatory duct. Secretions of the AGs, along with spermatophores from the seminal vesicle and spermatic fluid, enter into the female spermatheca via the ejaculatory duct during mating. Within the female spermatheca, the spermatophores are gradually broken down, and free spermatozoa are released to encounter oocytes, leading to fertilization [7]. In a previous study, we found that the AG secreted protein can effectively digest the spermatophore wall to release free spermatozoa and interact with proteins from the spermatheca to increase the acrosomal enzyme activity of free spermatozoa [8]. We also found that there are many small and large vesicles in the crab AG [9], that are visible under transmission and scanning electron microscopy. These vesicles are thought to contain the enzymatic proteins or other activation factors, which may be required for spermatophore rupture or other function in fertilization.

Recent advances in molecular imaging, genomic and proteomic technologies offer significant potential not only for gaining a better mechanistic understanding of *E*. *sinensis* reproduction, but also for achieving better aquaculture yields. Alternative strategies using multi-dimensional liquid chromatography separations coupled with tandem mass spectrometry (LC-MS/MS) have recently been applied to proteomic profiling of diverse organisms [10, 11, 12]. However, few studies have focused on the Chinese mitten crab, and none on its reproductive tissues. As many AG secreted proteins have not been structurally or functionally characterized in this animal, we identified them in *E*. *sinensis* using the highly efficient time- and labor-saving shotgun method. By utilizing SDS-PAGE to separate proteins by molecular weight, followed by in-gel digestion of proteins and LC-MS/MS analysis, we identified 486 proteins from the mature crab’s AG and established the first such reported experimental dataset for the AG proteome of the Chinese mitten crab, *E*. *sinensis*. This work lays a foundation for further studies of crustacean reproduction, as better knowledge of the crab reproductive biology is important for improving artificial control of breeding for commercial purposes and will contribute to the sustainable production and management of natural resources.

## Materials and Methods

### Protein extraction from the *E*. *sinensis* AG

Healthy sexually mature male Chinese mitten crabs (*E*. *sinensis*, 150–200 g) that had reached the stage of rapid AG development were obtained from a commercial aquaculture crab farm (Caojing Town, Jinshan District near Shanghai, China) in October, November and December of 2011. Male crabs were placed in an ice bath for 1–2 min until they were lightly anesthetized. Crabs were dissected on ice, and AGs were immediately removed. AGs tissues from three different individuals were taken on three occasions for the three different development stages, and these nine pairs of AGs tissue were pooled as a single sample for protein collection. AGs were excised in 5 mM Tris-HCl (pH 7.5) containing peptidase inhibitors (Complete, Mini, EDTA-free, Roche Diagnostics Ltd, Shanghai, China). The AGs were then centrifuged at 10,000 × *g* at 4°C to separate secreted AG proteins from the tissue. The supernatant was then removed and stored at-80°C until required. Protein samples were processed using a 2D clean-up kit (GE Healthcare, Shanghai, China). Total protein concentration was determined using a commercial kit based on the Bradford method according to the manufacturer’s instruction (Invitrogen, Shanghai, China).

### SDS-PAGE separation and in-gel tryptic digestion of proteins

**Fig 2:**
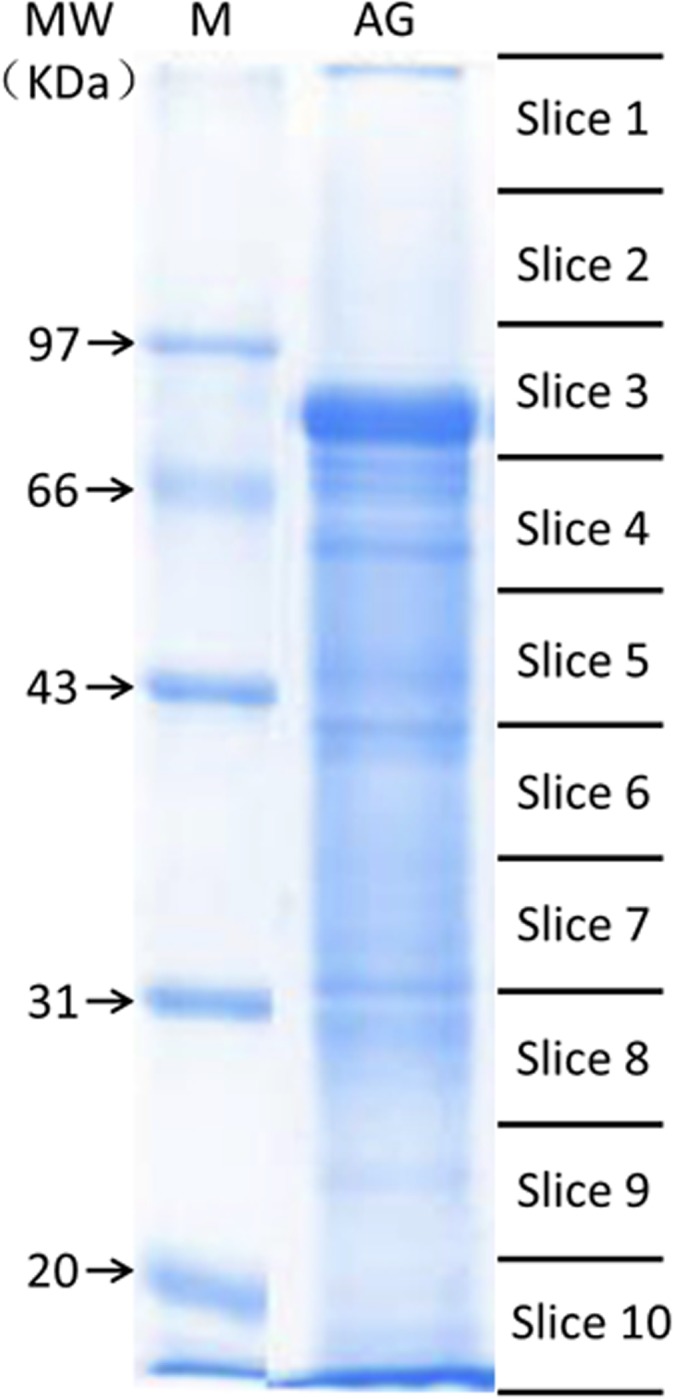
SDS-PAGE pattern of AG proteins in *E*. *sinensis*. AG proteins (30 μg) were separated using 10 cm 12% SDS-PAGE gels for 1 h at 120 V. The gel was stained with Coomassie Brilliant Blue G250 (Invitrogen) and the protein lane of the stained gel was cut into ten pieces of equal size.

AG proteins (30 μg) were solubilized in buffer (urea 7 M, thiourea, CHAPS 2%, SDS 4%, Tris 0.1 M pH 6.8, glycerol 30%, mercaptoethanol 12.5% and bromophenol blue 0.0062%) and separated using 10 cm 12% SDS-PAGE gels for 1 h at 120 V. The gel was stained with Coomassie Brilliant Blue G250 (Invitrogen) following the manufacturer’s instructions. The protein lane of the stained gel was cut into ten pieces of equal size (Fig 2). After being destained, the proteins were reduced in-gel, alkylated and digested with trypsin as previously reported [13]. Subsequently, each protein sample was analyzed by mass spectrometry.

### Nano-LC MS/MS analysis

Nano-LC MS/MS was performed on an HPLC system consisting of two LC-20AD nano-flow LC pumps, an SIL-20 AC auto-sampler and an LC-20AB micro-flow LC pump (all from Shimadzu, Tokyo, Japan) connected to an linear trap quadrupole (LTQ)-Orbitrap mass spectrometer (ThermoFisher, San Jose, CA). The sample was loaded onto a CAPTRAP column (0.5 x 2 mm, MICHROM Bioresources, Auburn, CA) over 3 min at a flow rate of 15 μL/min. The sample was subsequently separated by a C18 reverse-phase column (0.1 x 150 mm, packed with 3 μm Magic C18-AQ particles, MICHROM Bioresources) at a flow rate of 400 nL/min. The mobile phases were 5% acetonitrile with 0.1% formic acid (phase A and loading phase) and 95% acetonitrile with 0.1% formic acid (phase B). To achieve proper separation, a 90-min linear gradient from 5 to 45% phase B was employed. The separated sample was introduced into the mass spectrometer via an ADVANCE 30 μm silica tip (MICHROM Bioresources). The spray voltage was set at 1.0 kV and the heated capillary was kept at 180°C. The mass spectrometer was operated in data-dependent mode. Each cycle of duty consisted of one full MS survey scan at the mass range of 350~1800 Da with resolution power of 60,000 using the Orbitrap, followed by MS/MS analysis for the 10 strongest peaks using the LTQ. Peptides were fragmented in the LTQ section using collision-induced dissociation with helium, and the normalized collision energy value set at 35%. Only 2+ and 3+ peaks were selected for the MS/MS run, and previously fragmented peptides were excluded for 60 s.

### Protein database construction and searches

An in-house database was constructed with FASTA protein sequences downloaded from NCBI (http://www.ncbi.nlm.nih.gov/), including those of the crustacean suborder Pleocyemata (http://www.ncbi.nlm.nih.gov/Taxonomy/Browser/wwwtax.cgi?mode=Info&id=6692), along with the transcriptome data from *E*. *sinensis* published by He *et al*. [6] (208425 entries, 42305KB). Construction of the reference protein sequence databases included the target sequence, a decoy sequence and a contaminating-protein sequence. The target sequence is a valid protein sequence identified, the decoy sequence is the reverse of a given target sequence and is used to evaluate the results of the identification, and the contaminating-proteins sequence is a mix of the sequences of 248 contaminating proteins, a total of 150kb [14]. Protein searches were performed with Mascot 2.2.07 software (www.matrixscience.com) against the constructed database. The acceptance criterion for peptide identifications was a false positive identification rate of less than 1%. Database searches were performed with the following parameters: peptide mass tolerance of 20 ppm precision, charge state of 1+ and a maximum number of missed cleavages of 2. Carbamidomethylation of cysteines was used as a fixed modification and oxidation of methionine as a variable modification. Peptide identifications with a minimum peptide length of 6 amino acids, Mascot peptide identification score ≥60 and significant for peptide mass fingerprint with a p<0.05 were considered valid.

### Gene Ontology (GO) categories and Kyoto Encyclopedia of Genes and Genomes (KEGG) pathway analysis

The predicted subcellular locations and functions of the identified proteins were suggested by their GO component and function terms, respectively, which were downloaded as text-based annotation files from the GO database ftp site: ftp://ftp.geneontology.org/pub/go/ [15]. The query FASTA protein sequences were also searched against the KEGG GENES database using the BLASTP program with a BLOUSM62 scoring matrix (http://blast.genome.jp/). The Enzyme Commission (EC) number (if available) of the best matched protein (E-value < e^-15^) was accepted and exported. The obtained EC numbers from each dataset were subjected to a pathway search against the KEGG reference pathway database (http://www.genome.jp/kegg/tool/search_pathway.html). Each selected pathway met the criterion of having at least three ECs. Pairwise comparison of the pathways were undertaken and then classified according to the KEGG definition (http://www.genome.ad.jp/kegg/pathway.html).

### Validation of Es-serpin function as a decapacitation factor

Approximately 35–40g of ovaries were harvested from female crabs that were beginning to ovulate (about 300g), and then cut them into pieces. The shredded ovaries were soaked in about 200ml of pre-cooling artificial seawater for 24 hours at 4°C. Then the supernatant (“eggs water”) was used to induce the acrosome reaction (AR). The AR was induced by eggs water in the dark at room temperature in a 1.5-mL Eppendorf tube. For indirect immunofluorescence studies of Es-serpin in sperm were prepared according to Ye Bi et al. [16]. The reaction was then terminated with 4% paraformaldehyde at 10 min, 20 min, 30 min, and 40 min. Sperm samples were then washed and fixed with 4% paraformaldehyde (W/V) for 30min, permeabilized with 1% Triton X-100 in PBS for 15 min on ice (this step was omitted from the indirect immunofluorescence experiments with non-permeabilized sperm), followed by blocking with 10% BSA at room temperature for 1 hr. After incubation with primary antisera (1:500) overnight at 4°C, the fixed sperm were incubated with fluorescein isothiocyanate (FITC; Beijing Zhongshan Biotechnology Co.)—conjugated anti-Rabbit IgG at a dilution of 1:100 for 1h at room temperature. Finally, DAPI (4’,6-diamidino-2-phenylindole; Beijing Zhongshan Biotechnology Co.) was applied for 5 min without subsequent washing. The samples were then applied to slides, mounted in ~100uL antifade mounting medium (Beyotime Institute of Biotechnology) and photographed using a fluorescence microscope (Leica DM4000 B LED).

## Results and Discussion

### Protein identification and annotation

We report the first successful identification of AG secreted proteins from *E*. *sinensis*. Mass spectra of a total of 1173 peptides generated (1067 without decoy and contaminants) allowed for the identification of 486 different proteins (S2 and S3 Tables). The average length of the peptides and proteins were 15 aa and 273 aa, respectively. The mass spectrometry proteomics data have been deposited to the ProteomeXchange Consortium (http://www.proteomexchange.org) via the PRIDE partner repository [17] with the dataset identifier PXD000700.

### GO and pathway annotation

**Fig 3:**
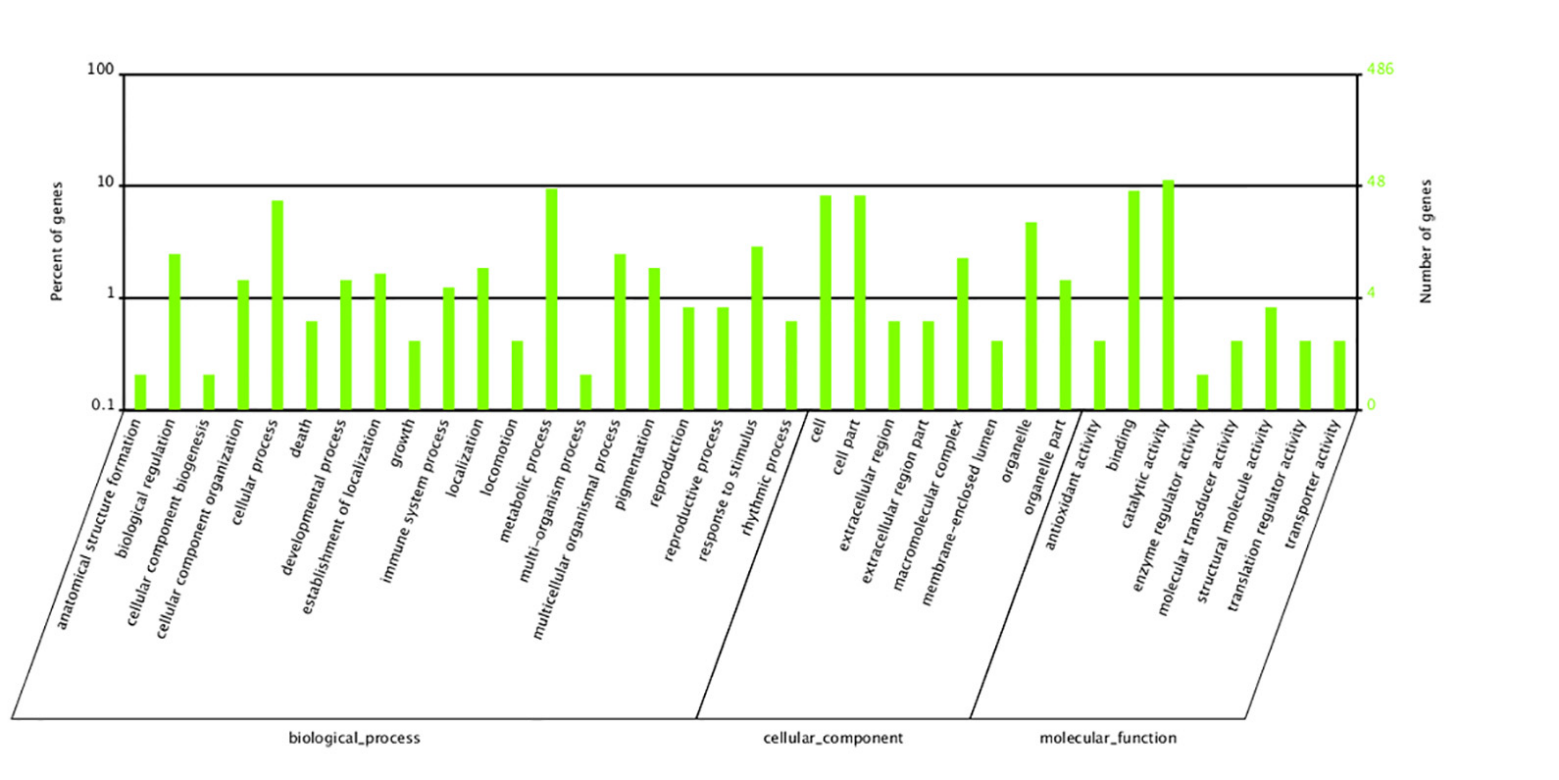
GO annotation of AG proteins in *E*. *sinensis*. The subcellular locations and functions of the identified proteins were determined by their GO component and function terms, respectively, available for download as text-based annotation files from the GO database ftp site: ftp://ftp.geneontology.org/pub/go/

To help determine the potential function of the 486 proteins identified by LC-MS/MS, we performed GO analysis based on their predicted GO hierarchy or on the GO hierarchy of similar proteins annotated in other species [18]. GO protein classification is based on three different categories: biological process, cellular component and molecular function, with 20, 8 and 8 subsets, respectively. Some of the *E*. *sinensis* AG proteins fell into included in more than one category. The cellular component category included 129 proteins most of which fell into three subgroups: those from the cell (31.0%), the cell part (31.0%) and organelles (17.8%). Based on the annotated molecular functions, most of the proteins identified possessed predicted catalytic activity (49.1%) or protein binding capabilities (39.3%). Within the biological process category, the greatest percentages of proteins were involved in “metabolic processes” (24.6%) and “cellular processes” (19.3%). Only 4.3% of the proteins were previously annotated as being related to reproduction (Fig 3). Since there has been little basic research on the Chinese mitten crab and there is no genome sequence information for this animal, proteomic data analysis is limited, but finding such as ours provide a basic protein dataset for reproductive study in this species.

Biochemical pathway information was collected by downloading relevant maps from the KEGG database (http://www.genome.jp/kegg/) [19]. A total of 394 proteins were associated with 150 predicted KEGG metabolic pathways, and the number of different pathways ranged from 1 to 125 (S4 Table). The top pathways with highest protein numbers were metabolic pathways (125, 31.73%), pathogenic *Escherichia coli* infection (27,7.11%), phagosome (25, 6.35%), proteasome (24, 6.09%), tyrosine metabolism (22, 5.58%) and protein processing in the endoplasmic reticulum (ER) (21, 5.33%). Finding these pathways represented suggests that the reproductive related MAPK signaling pathway, Wnt signaling pathway and Notch signaling pathway all exist in *E*. *sinensis* and suggests that their further analysis is warranted; our results also provide the first step to allow this analysis.

### AG proteins related to reproductive processes

Protein components of the seminal fluid play significant roles in a wide range of sexual reproductive phenomena. Recent studies have shown an increasing interest in obtaining proteomic information on both male reproductive glands from different species [10,20,21], but detailed proteomic profiles at the individual tissue level have not be reported for *E*. *sinensis*. Male gametes are produced in the testes, and mature sperm are stored in the seminal vesicles until ejaculation. Components of male seminal fluids of *E*. *sinensis* are secreted by the vas deferens and AGs. Each secreted seminal component plays a distinctive role in the success of reproduction. The most relevant proteins identified in the AG involved in reproduction or in the interaction with spermatozoa according to the literature are shown in S1 Table. These proteins fall into five main groups: protease and protease inhibitors, folding and stress response proteins, crustacean molting protein, signaling pathway factors and structural and ubiquitin system proteins function in reproductive system. Below, we discuss these five categories in the context of understanding AG function in of *E*. *sinensis* reproduction (Table 1).

**Table 1:** Important proteins function in reproduction in *E*. *sinensis* (selected).

Protein name	Peptides	Species	PEP	Score	Sequence Length	Theor. MW(kDa)	Peptides	Unique Peptides	Sequence coverage(%)
**Enzymes (catalytic activity)**									
serine proteinase inhibitor 6	AEAQPFYCNRPFIFLIYDEDTK(13)	*Penaeus monodon*	8.67E-07	144.3(13)	48	5.4503	2	2	64.6
	VVLFVGAYK(1104)			159.04(1104)					
protein-disulfide isomerase	GFPTIFWK(337)	*Scylla paramamosain*	1.42E-14	104.21(337)	286	32.288	3	3	16.1
	MDATANDVPEAFNVR(697)			242.07(697)					
	TQDTASEFVPPLVVAYFNVDYVK(977)			157.57(977)					
Protein phosphatase 2A, catalytic subunit, β1	LQEVPHEGPMCDLLWSDPDDR(640)	*-*	3.96E-49	162.65(640)	258	29.646	3	3	19.4
	QITQVYGFYDECLR(799)			249.32(799)					
	SPDTNYLFMGDYVDR(897)			344.54(897)					
pyruvate kinase 3	AEVSDVGNAIMDGADCVMLSGETAK(20)	*Litopenaeus vannamei*	5.74E-40	200.74(20)	229	24.774	4	4	27.1
	GDYPLVCVR(324)			133.23(324)					
	MGVDMVFASFIR(706)			155.97(706)					
	NIDSIIEEGDGIMIAR(737)			312.49(737)					
GDP mannose-4,6-dehydratase	ALVTDMMQADIDLMR(63)	*Tribolium castaneum*	1.48E-32	284.24(63)	94	10.805	2	2	30.9
	EIYWEGSGVDEVAK(211)			280.21(211)					
Inorganic pyrophosphatase	VDLWHYVALK(1014)	*Harpegnathos saltator*	5.55E-09	208.22(1014)	100	11.138	2	2	26
	VIMETHESWQHLVEGK(1042)			210.64(1042)					
cystathionine-beta-synthase	EGYDQLPVVDQEGMIR(199)	*Xenopus (Silurana)*	1.63E-10	165.86(199)	386	42.657	2	2	11.1
	VSTLHLPAPLTVLPTIACQDAIAIMQR(1091)	*tropicalis*		157.88(1091)					
**Glycolytic enzymes**									
transaldolase	ALGGCDLLTIGPK(50)	*Aedes aegypti*	1.58E-26	201.17(50)	298	33.553	3	3	15.8
	FLEELQNSTEPVVQHLSEASAK(274)			176.81(274)					
	ILDWYVANTDQK(471)			275.76(471)					
fructose 1,6-bisphosphatase	AGIAQLYGMAGDVNVQGEEVK(30)	*Marsupenaeus japonicus*	1.33E-81		313	33.795	8	8	33.5
	KAGIAQLYGMAGDVNVQGEEVK(517)								
	LLYECNPMAFLVQQAGGK(615)								
fructose-6-phosphate aminotransferase	GALIVGITNTVGSSICR(312)		1.09E-19		285	31.284	4	4	16.5
	GYNYATCLEGALK(394)								
	YASSVLELPK (1125)								
UDP-glucose 4-epimerase	AVGESMQFPLIYYK(92)		1.34E-07		85	9.7071	3	3	48.2
	IDYVIHFAAMK(435)								
	VTFYQCDLLDGQTVHK(1095)								
**Crustacean molting protein**									
cryptocyanin	DGNGAIIPFDEGR(125)	*Metacarcinus magister*	4.85E-109		653	75.767	13	4	15.8
	LLAMPYRDGNGAIIPFDEGR(594)								
	TLVEKTENR(964)								
cryptocyanin 2	ERVNEEEFIYAAYHAVK(229)	*Metacarcinus magister*	4.57E-127		674	78.26	13	3	14.8
	RPFGYPLDR(838)								
	VNEEEFIYAAYHAVK(1064)								
beta-1,3-D-glucan binding protein	IESSLTPLPALVFGLGR(440)	*Pacifastacus leniusculus*	8.53E-42		271	30.24	8	8	33.6
	LNEFIFEMHNHFER(624)								
	LYVEVHGESSFELVR(687)								
similar to past-1	IGPEPTTDGFIAVMYGDTER(457)	*Nasonia vitripennis*	4.83E-16	226.74(457)	227	25.796	3	3	18.1
	IILLFDAHK(461)			123.69(461)					
	VYIGSYWNEPLR(1110)			199.03(1110)					
ferritin 3	AGISGLGEFLFDK(31)	*Eriocheir sinensis*	1.96E-08	164.81(31)	170	19.573	3	3	26.5
	AGISGLGEFLFDKEFE(32)			203.32(32)					
	IVLQAIAAPPQQEWGNCNDALQAALDLEK(511)			74.222(511)					
alpha 2-macroglobulin	AYALALAERPEAMVVLNELMAR(97)	*Eriocheir sinensis*	3.50E-82		1457	162.1	13	10	12.8
	GMLNIPITLPFSLHSVTPLAR(366)								
	VLVWLVRPDGEVVADAR(1059)								
peroxiredoxin	ALNAEVVACSIDSHFTHLAWTNTPR(58)	*Penaeus monodon*	2.55E-21		235	26.241	4	4	23
	IPLLSDITHK(493)								
	QITMNDLPVGR(798)								
**Signal transducers**									
Ras-related protein Rab-1A	GAHGIIVVYDTTDQESFNNVK	*Scylla paramamosain*	1.31E-103	220.27	132	14.501	3	1	40.9
eukaryotic initiation factor 4A	FLPSEVQVVLLSATMPSDVMDVTTK(281)	*Callinectes sapidus*	7.24E-43		432	48.725	8	8	26.2
	GIDVQQVSLVINYDLPTNR(353)								
	TSIEEMPMNVADLI(979)								
ADP ribosylation factor 4	DAVLLVFANK(117)	*Marsupenaeus japonicus*	1.61E-22	193.82(117)	70	8.002	3	3	45.7
	LGLNQLR(573)			154.11(573)					
	QDLPNAMTAAELTDR(787)			271.64(787)					
receptor for activated protein kinase c1	LWDLAAGK(683)	*Penaeus monodon*	0.0001362	108.46(683)	99	11.305	2	2	19.2
	NFPDMILSASR(735)			162.26(735)					
muscle elongation factor 1 gamma	DPLDAFPAGNFNMDDFKR(159)	*Procambarus clarkii*	6.97E-09	148.03(159)	173	20.19	2	2	18.5
	IFMSCNLIGGMFQR(446)			223.22(446)					
**Transporters and protein trafficking**									
importin beta-3	FEPYLPLVMGPVLK(254)	*Culex quinquefasciatus*	0.0001007	174.05(254)	326	36.193	2	2	10.4
	LVLEQVVTTIASVADTAEEK(677)			83.894(677)					
**Defense molecules**									
ubiquitin carboxyl-terminal esterase L3	AHEESAQEGQTEAPDR(38)	*Scylla paramamosain*	1.81E-10	119.96(38)	126	14.131	3	3	42.1
	EAQVNEHFVAFVHVDGK(179)			138.21(179)					
	QFVGNACGTVALIHAIANNR(792)			197.75(792)					
20S proteasome alpha subunit	ITSPLMIPSTIEK(506)LFQVEYAIEAIK(559)	*Scylla paramamosain*	5.81E-09	160.67(506)	103	11.366	2	2	24.3
				201(559)					
**Chaperone molecules**									
glucose-regulated protein 78	ITPSYVAFTADGER(505)	*Fenneropenaeus chinensis*	3.26E-54		352	39.331	7	4	28.7
	TVTHAVVTVPAYFNDAQR(1001)								
	VFAAEEVSAMVLGK(1023)								

Note: PEP (Posterior error probability) is the probability of an individual match.

#### Proteases and protease inhibitors

Approximately 18% of the proteins in the *Drosophila melanogaster* ejaculate are predicted proteases or protease inhibitors. LaFlamme *et al*. found that proteases in the seminal fluid can destroy, activate or otherwise modulate the function of other proteins [4]. Several seminal proteins in this organism are activated or degraded after mating, and proteolysis is an effective way to accomplish this process since these proteins act outside of the cell where most other regulatory processes cannot be used. Proteases and protease inhibitors are known to be major constituents of the male seminal fluid, and RNAs encoding 120 predicted proteases and 8 protease inhibitors were identified in our previous AG transcriptome analysis in *E*. *sinensis* [6]. During epididymal transit, sperm bind new proteins, and proteins already present in the membrane are modified [22]. Enzymes in addition to proteases also likely play key roles in membrane transformations, and several of these were detected in the present study, including cathepsin A, B, protein-disulfide isomerase, inorganic pyrophosphatase, AICAR transformylase, cystathionine-beta-synthase and cytoplasmic manganese superoxide dismutase.

**Fig 4:**
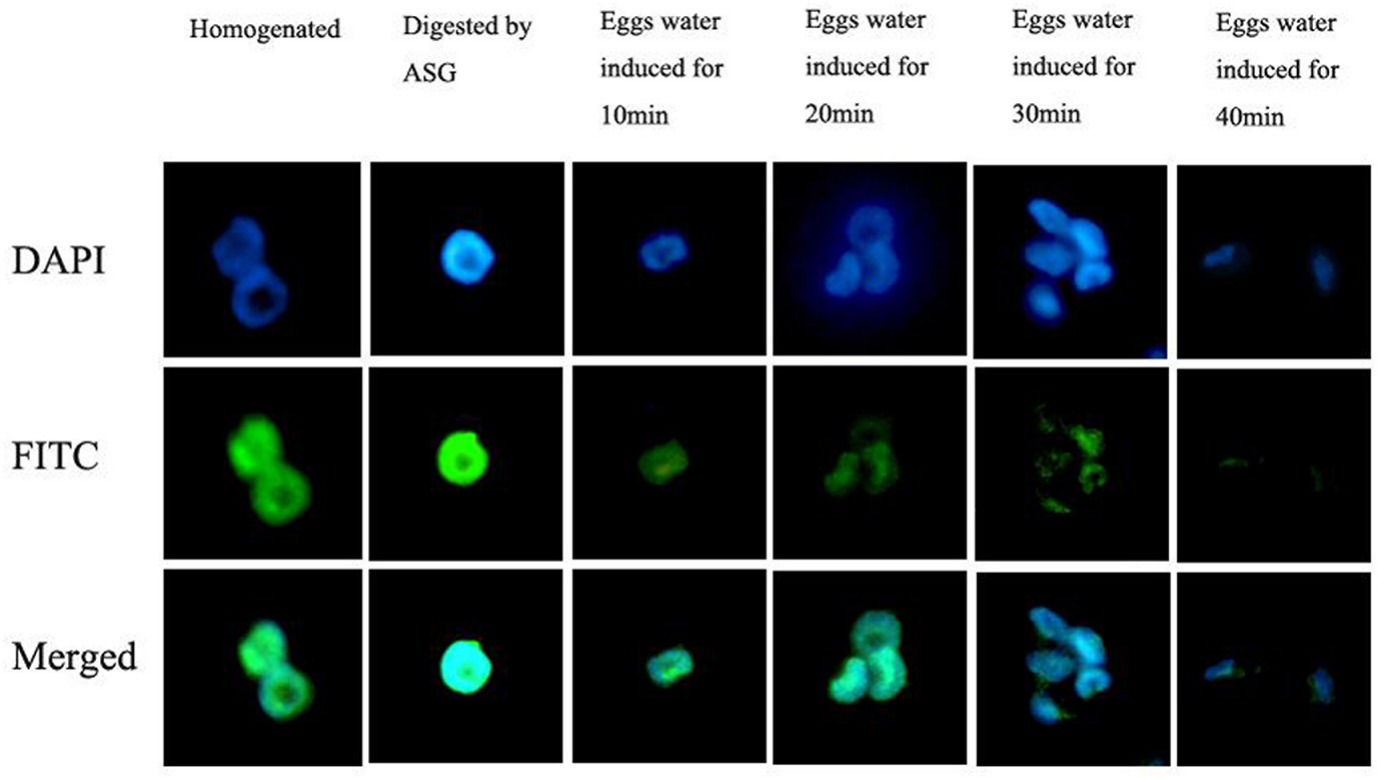
Changes in immunofluorescence staining patterns of antibodies against human Es-serpin in *E*. *sinensis* sperm during the acrosome reaction. Sperm were incubated in eggs water for 40min, and the reaction was terminated by 4% paraformaldehyde respectively at 10min, 20min, 30min, 40min.

In the mammalian cauda epididyma, protease activity is modulated by protease inhibitors. We identified several predicted protease inhibitors among *E*. *sinensis* AG proteins. These included predicted SERPINs (serine protease inhibitors), glycoprotein, pacifastin and serine collagenase 1 precursor. We propose that such components of the seminal fluid are likely important for regulating proteolysis for essential processes or to preserve sperm and tissue integrity [23]. Proteins in mammal seminal fluid include serpin 6 and HongrES1, which functions as a decapacitation factor (DF) for sperm, preventing capacitation. HongrES1 is specifically expressed in the cauda epididymis of rat and guinea pig [24,25] where it is important for fertility. Given our findings of predicted serpins in Chinese mitten crab AGs, we used antibodies to HongrES1 to test if there is similar protein associated with sperm of Chinese mitten crab. We further examined the amount of this Es-serpin on sperm before and after the AR. The results (Fig 4) showed that after the occurrence of AR, Es-serpin cross reactivity on the sperm surface gradually decreased. This is consistent with the variation of DF in sperm, and suggests that we may have identified a DF in *E*. *sinensis*.

Other protease inhibitors that we detected included Pacifastin, which is a serine peptidase inhibitor [26], that is found in insects and arthropods. Some studies have shown that crustacean pacifastin plays a key role in the immune response, whereas insect pacifastin-like peptides have multiple regulatory functions including ones related to immunity, reproduction and phase transition [27]. There are five additional members (SGPI-1–5) of this family from ovarian tissue of the desert locust *Schistocerca gregaria*. All of these peptides possess the cysteine pattern that is characteristic of the pacifastin light chain domains (PLD 1–9) and the locust inhibitors PMP-D2, PMP-C and HI. Further, there is evidence that transcripts of these protease inhibitors are differentially regulated during the molting and reproductive cycles of the locust, suggesting that the encoded peptides have distinct physiological roles in a variety of physiological processes [28]. Previously the mRNA expression of the *pacifastin* gene of *E*. *sinensis* was analyzed in different tissues of the crab [29]. Here, we identified the pacifastin protein in extracts from the AG, implying that it may also have a reproductive function in *E*. *sinensis*. Further analyses using mutants or RNAi will provide insights into the function and physiological roles of these proteins in crustacean reproduction.

Glycolytic enzymes detected in the present study include predicted glutamate dehydrogenase, alpha glucosidase 2, fructose 1, 6-bisphosphatase, fructose-6-phosphate aminotransferase and UDP-glucose 4-epimerase. These enzymes may hydrolyze sugar moieties present on membrane glycoproteins during epididymal sperm maturation and thus potentially mediate sperm/oocyte interactions [30], they could also hydrolyze sugar moieties on Sfps or even on female reproductive tract proteins, thus potentially playing roles in sperm maturation and physiological regulation of fertilization in crustaceans.

#### Protein folding and stress response proteins

We identified some chaperone molecules such as predicted heat shock protein in the AG proteome. Most of these types of proteins function in protein folding or stress response reactions. Glucose-regulated protein 78 (GRP78), a member of the heat shock protein 70 family, is an ER-resident protein responsible for protein folding and assembly [31]. The 94-kDa glucose-regulated protein (GRP94), also known as endoplasmin, 96-kDa glycoprotein (GP96) or HSP108, belongs to the HSP90 family, is an ER chaperone and was first identified in a screen for proteins influenced by glucose starvation. GRP94 mainly localizes to the ER and is thought to be required for the folding and maturation of secretory and membrane proteins [32]. Therefore, the presence of these molecular chaperones among AG proteins suggests that they play roles in aiding the correct folding and assembly of proteins in the AG, and protecting them from stress responses. Endoplasmic reticulum protein 29 (Erp29) plays an important role in the processing of secretory proteins within the ER, possibly by participating in the folding of proteins [33]. HSP70–3 is a BiP homolog involved in assembling protein complexes inside the ER [34]. Indeed, recent studies have shown that cell-surface chaperone proteins can have important roles in reproduction by functioning as intercellular signaling molecules that facilitate sperm-oocyte interactions [35].

Two other interesting proteins that we found were a predicted catalase and a predicted cyclophilin. These are interesting to find in AG secretions. Maturation of sperm involves membrane remodeling and changes in sperm metabolism. For example, when sperms are stored in the cauda segment of the epididymis in mammalian, they must be protected against oxidative/peroxidative reactions, immune attack and damage caused by substances released by dying cells [10]. Catalase serves to protect against toxic effects of hydrogen peroxide by promoting growth of cells, including T-cells, B-cells, myeloid leukemia cells, melanoma cells, mastocytoma cells and normal and transformed fibroblast cells [36]. Here, we identified a predicted catalase in the AG proteome from *E*. *sinensis*.

Cyclophilins possess a peptidyl-prolyl cis-trans isomerase (PPIase) activity that rotates the polypeptide chain around a proline between a cis and a trans configuration. They have multiple roles in the folding, assembly and trafficking of cellular proteins [37]. Although considered to be primarily intracellular proteins, some mammalian cells secrete cyclophilins into the extracellular environment, where they have an important role in mediating cellular responses to oxidative stress and in modulating immunity. We also identified cyclophilin A protein in our dataset.

#### Proteins associated with crustacean molting protein

Crustaceans possess a calcified exoskeleton that is replaced during growth and development by molting, which occurs periodically under the control of neuropeptide hormones and ecdysteroids. We previously identified 64 unigenes related to hormones in our transcriptome analysis of the testis and AG [6]. Some crustacean molting related proteins in the AG proteome were also identified and will be discussed below.

Cryptocyanin, a crustacean molting protein, has an evolutionary link with arthropod hemocyanins and insect hexamerins [38]. In the Dungeness crab, *Cancer magister*, hemocyanin reversibly binds oxygen at its highly conserved copper-oxygen binding sites and supplies the crab’s tissues with oxygen. Expression analysis of hemocyanin subunits from the megalopa to adult crab have indicated that there are dramatic changes in subunit composition and oxygen affinity that are correlated with developmental changes in salinity tolerance and ion regulation[39,40]. Cryptocyanin closely resembles hemocyanin in sequence, but lacks several critical copper-binding amino acids and has lost the ability to bind oxygen. Its presence in high concentrations, particularly at specific times in the molt cycle, suggests it has been exploited to carry out new functions. Studying the dynamics of synthesis of hemocyanin and cryptocyanin at the molecular, tissue and organismal levels, including differential responses to hormonal regulation will be an interest area in crustaceans. In this study we identified two cryptocyanin proteins and one hemocyanin protein among secreted AG proteins of male *E*. *sinensis*.

Marine invertebrates demonstrate a rich diversity in form and function of their oxygen transport proteins, reflecting how the evolution of new proteins resulting from gene duplications and subsequent mutations is an important process in the adaptive ability of organisms. Phenoloxidase, a third member of the oxygen-transport protein family, functions in sclerotization of the new exoskeleton after molting as well as in immune defense. Its copper-oxygen binding sites are similar to those of hemocyanin, but it is an intracellular protein that is found in circulating hemocytes [41]. Hemocyanin itself has phenoloxidase activity, along with potential anti-microbial activity, which suggests additional functions in response to environmental stressors. The quaternary structure of hemocyanin affects its reactivity as phenoloxidase. It was suggested that prophenoloxidase is released from hemocytes and moves across the epidermis into new exoskeleton during the pre-molting stage and is activated in the early post-molting stage [42]. In crustaceans and insects, 1,3-P-glucan binding proteins (PGBPs) have been purified from plasma [43] and are also found in crustacean blood. These proteins enhance the glucan-mediated activation of the prophenoloxidase activating system (proPO-system) [44]. Vitellogenin may serve one of two functions, as a yolk protein precursor in its classical role and potentially to shuttle iron to developing germ cells.

#### Functions of signaling pathway factors

Signaling molecules like phospholipase A, calcium calmodulin, phosphatidylinositol 3-kinase, MAPK signaling pathways and Wnt signaling pathways have been associated with cell proliferation and differentiation as well as with metabolic processes in mammalian granulosa and Sertoli cells [45]. Here, we identified three MAPK signaling pathway proteins (Rap1, FLNA and HSP72) and two Wnt signaling pathway proteins (PP2A and Rho A) among AG proteins of *E*. *sinensis*. ERKs and p38 have been demonstrated to be involved in the acrosome reaction and function in spermatogenesis. We also validation the function of p38 in acorosome reaction in sperm of *E*. *sinensis* [46]. Gpr48 (G protein-coupled receptor 48) participates in the development of the male epididymis and efferent ducts through regulation of ERα expression via the cAMP/PKA signaling pathway [47].

The 14–3–3 protein positively regulates Ras-mediated pathways and acts downstream or parallel to Raf, but upstream of nuclear factors in Ras signaling [48]. The fibrillarin protein, involved in pre-rRNA processing, is a component of several ribosomal [49] and nucleolar protein complexes and interacts with DDX5 (belonging to the DEAD box protein family) [50]. In mammals, past-1 is highly expressed in the testis and acts in early endocytic membrane fusion and membrane trafficking of recycling endosomes [51]. A recent report demonstrated that the single *Drosophila* EHD ortholog Past1 is expressed ubiquitously during early embryogenesis, exhibits both plasma membrane associated and punctate cytosolic staining, and is capable of binding *in vitro* to the adaptor protein Numb [52]. Numb is a conserved membrane-associated protein that antagonizes Notch signaling, a pathway which carry out important functions during oogenesis as well as spermatogenesis [53].

ADP-ribosylation factors (ARFs) are small GTP-binding proteins that play essential roles in intracellular trafficking and organelle structure. ARFs were originally identified as a protein cofactor involved in membrane transport, maintenance of organelle structures and remodeling of the cytoskeleton [54]. Although all ARFs have the ability to regulate the budding and formation of vesicles in the endocytic and exocytic pathways, different ARFs seem to possess different intracellular localizations and functions depending on the specific membrane to which it binds by virtue of recruitment of diverse proteins. Recently, Ran, Rab and Rho proteins, small GTPases in shrimp, were reported to be involved in host defense responses [55]. ARF GTPases are well known to play critical roles in intracellular vesicular trafficking, signal transduction, phagocytosis, endocytosis and remodeling of the cytoskeleton [56]. Here, we identified an ADP ribosylation factor 4 in the *E*. *sinensis* AG proteomic analysis. The ecto-ADP-ribosyltransferase 5 (ART 5) protein identified in the AG belongs to a class of enzymes that catalyze the transfer of an ADP-ribose group from NAD to arginine residues on target proteins, such as G proteins, rho, actin, CD44 and integrins [57]. ADP-ribosyltransferases (ARTs) are normally expressed in immune cells and ART5, in the testis [58].

#### Structural and ubiquitin system proteins

The heparan sulfate proteoglycan core protein is an integral component of the glomerular basement membrane (GBM), which is responsible for the fixed negative electrostatic membrane charge and provides a barrier that is both size- and charge-selective. It serves as an attachment substrate for cells playing essential roles in vascularization, such as normal heart development, regulating the vascular response to injury and avascular cartilage development [59]. We identified a related protein (Basement membrane-specific heparan sulfate proteoglycan core) in our proteomic analysis, but further analysis is required to determine the function of this protein in AG.

Actin is involved in the initiation of sperm motility, and during epididymal storage in mammals it forms a barrier preventing a premature acrosome reaction [60]. Profilin binds to actin and affects the structure of the cytoskeleton. At high concentrations, profilin prevents the polymerization of actin, whereas it enhances it at low concentrations. By binding to PIP2 (Plasma membrane intrinsic protein 2), profilin inhibits the formation of IP3 (inositol 1,4,5-triphosphate) and DG (diacylglycel). Profilin also can inhibit the androgen receptor (AR) and huntingtin aggregation, and binding of G-actin is essential for its inhibition of AR [61].

In *Drosophila* reproduction, lectin and cysteine-rich secretory proteins (CRISP) and lipases may play roles in providing energy to the sperm by producing free fatty acids for β-oxidation [62]. Our proteomic analysis detected a predicted lectin in *E*. *sinensis*. The laminin receptor can bind to cells via a high affinity receptor, and laminin is thought to mediate the attachment, migration and organization of cells into tissues during embryonic development by interacting with other extracellular matrix components. Fragments of laminin chains can act as biologically active peptides to perturb the blood–testis barrier (BTB) permeability function by accelerating protein endocytosis (e.g., occludin) at the site, thereby destabilizing the BTB integrity to facilitate the transit of preleptotene spermatocytes [63]. Our identification of a predicted laminin receptor in the *E*. *sinensis* AG proteome suggests that laminin may be involved in such a process in this crab.

The coatomer is a cytosolic protein complex that binds to dilysine motifs and reversibly associates with Golgi non-clathrin-coated vesicles, which further mediate biosynthetic protein transport from the ER, via the Golgi up to the trans Golgi network [64]. The coatomer protein complex (COP) is required for budding from Golgi membranes and is essential for the retrograde Golgi-to-ER transport of dilysine-tagged proteins. The complex also influences the Golgi structural integrity, as well as the processing, activity and endocytic recycling of LDL (low-density lipoprotein) receptors [66]. It is involved in the Golgi disassembly and reassembly processes during the cell cycle and in autophagy by playing a role in an early endosome function [65]. Kitazawa *et al*. suggested that COP plays an important role in *Drosophila* male meiosis, not only through vesicle transport to the cleavage furrow region, but also through the formation of ER-based structures [67]. Required for limiting storage in lipid droplets, COP is involved in lipid homeostasis by regulating the presence of perilipin family members PLIN2 and PLIN3 at the lipid droplet surface. Furthermore, it promotes the association of adipocyte triglyceride lipase (PNPLA2) with the lipid droplet surface to mediate lipolysis, suggesting a relevant role in providing energy for reproduction. Interestingly, we identified four coatomer subunits in the *E*. *sinensis* AG proteome.

We also identified components of the ubiquitin proteasome system in the AG proteomic analysis, including 24 proteasome pathway proteins and 2 ubiquitin mediated proteolysis proteins (UBE1 and UBE2D_E). The omnipresent ubiquitin–proteasome system (UPS) is an ATP-dependent enzymatic machinery that targets substrate proteins for degradation by the 26S proteasome by tagging them with an isopeptide chain composed of covalently linked molecules of ubiquitin, a small chaperone protein. UPS has been suggested to play a role in fertilization in humans and some other animals during the process of sperm penetration through the egg’s vitelline coat (VC) [68]. In ascidians, spermatozoa release ubiquitin-activating and conjugating enzymes, proteasomes and unconjugated ubiquitin to first ubiquitinate and then degrade the sperm receptor on the VC. In echinoderms and mammals, the VC [zona pellucida (ZP) in mammals] is ubiquitinated during oogenesis and its sperm receptor is degraded or inactivated during fertilization. Various proteasome subunits and associated enzymes have been detected in spermatozoa and localized to sperm acrosome and other sperm structures [69]. Fertilization is the process whereby individual gametes from the female (egg) and male (sperm) unite to produce offspring. Interaction with the egg is restricted to the sperm head, which contains a large secretory vesicle, the acrosome, overlying the sperm nucleus. Exocytosis of the acrosome (during the AR) is a terminal morphological alteration that must occur prior to penetration of the extracellular coat of the egg (ZP). As is the case with regulated exocytosis in somatic cells, Ca^2+^ is an essential mediator of the AR [70].

## Conclusions

The proteomic profiles of AG provide us with information about reproductive proteins in *E*. *sinensis* and supply the basis for future studies. This is the first report of AG protein identification, annotation and GO pathway analysis in *E*. *sinensis*. Even if the identification method and dataset only reflect the mature stage of AG, but the clues give us informative candidates for study their function in sperm maturation and fertilization. The candidate proteins identified here will be interesting candidates for further functional studies for their roles in reproduction of *E*. *sinensis*, Of particular interest are the proteases and protease inhibitors and the proteins associated with crustacean molting, sperm capacitation and acorosome reaction. Functions are known for some seminal fluid proteins in some animals. For example, in Drosophila or in mammals, specific seminal proteins have been shown to influence, female sperm storage, ovulation and post-mating behaviors. It will be interesting to determine the function of individual SFPs in sperm binding in reproduction of *E*. *sinensis* in the future.

## Supporting Information

S1 Table Important protein functions in reproduction in *E*. *sinensis*. (PEP is the probability of an individual match).
(DOC)

S2 Table Identified peptides in AG proteomics from *E*. *sinensis*. (XLS)

S3 Table Identified protein Groups in AG proteomics from *E*. *sinensis*. (XLS)

S4 Table KEGG analysis for AG proteomics from *E*. *sinensis*. The Enzyme Commission (EC) number of the best match protein (E-value < e^-15^) was accepted and exported.
(DOC)
