# Supplementary material for: A Catalog of Proteins Expressed in the AG Secreted Fluid during the Mature Phase of the Chinese Mitten Crabs (Eriocheir sinensis)
Source: PLoS One. 2015 Aug 25;10(8):e0136266. doi: 10.1371/journal.pone.0136266 (PMC4549300; doi:10.1371/journal.pone.0136266)
Supplement: S1 Table — (PEP is the probability of an individual match). (DOC) [file pone.0136266.s001.doc]

| Protein name | Peptides | Species | PEP | Score | Sequence Length | Theor.  MW(kDa) | Peptides | Unique Peptides | Sequence coverage（％） |
| --- | --- | --- | --- | --- | --- | --- | --- | --- | --- |
| **Enzymes (catalytic activity)** | |  |  |  |  |  |  |  |  |
| cathepsin A | FTNLDLVTVR | *Eriocheir sinensis* | 3.87E-05 | 211.78 | 465 | 52.531 | 1 | 1 | 2.2 |
| cathepsin B | HGLPLGGHAIR | *Marsupenaeus japonicus* | 0.01995 | 123.35 | 128 | 14.325 | 1 | 1 | 8.6 |
| Serine protease inhibitor | DLFVSNVVHK | *Branchiostoma lanceolatum* | 0.0034551 | 173.37 | 51 | 5.4861 | 1 | 1 | 19.6 |
| serine proteinase inhibitor 6 | AEAQPFYCNRPFIFLIYDEDTK(13)  VVLFVGAYK(1104) | *Penaeus monodon* | 8.67E-07 | 144.3(13)  159.04(1104) | 48 | 5.4503 | 2 | 2 | 64.6 |
| serine collagenase 1 precursor | VTYFLDWIQTHTGVTP | *Celuca pugilator* | 0.0022019 | 160.48 | 37 | 3.9223 | 1 | 1 | 43.2 |
| serpin 6 precursor | FTADHPFFFYIR | *Branchiostoma lanceolatum* | 0.00010191 | 185.08 | 65 | 7.4514 | 1 | 1 | 18.5 |
| pacifastin | GICTTLACLPSER | *Eriocheir sinensis* | 1.44E-05 | 207.88 | 276 | 30.042 | 1 | 1 | 4.7 |
| glycoprotein | LSDSPCALVASMFGWTGNMER | *Tribolium castaneum* | 2.29E-05 | 162.26 | 58 | 6.4503 | 1 | 1 | 36.2 |
| dehydrogenase,glyceraldehydephosphate | IGIDGFGR | *-* | 6.94E-16 | 127.12 | 333 | 35.717 | 3 | 1 | 9.9 |
| phosphoenolpyruvate-carboxykinase | VLDWILR | *Neohelice granulata* | 0.019468 | 121.29 | 646 | 72.129 | 1 | 1 | 1.1 |
| arginyl-tRNA synthetase | LVDLLNEGLQR | *Apis mellifera* | 3.26E-05 | 202.56 | 346 | 39.661 | 1 | 1 | 3.2 |
| protein-disulfide isomerase | GFPTIFWK(337)MDATANDVPEAFNVR(697) TQDTASEFVPPLVVAYFNVDYVK(977) | *Scylla paramamosain* | 1.42E-14 | 104.21(337)  242.07(697)  157.57(977) | 286 | 32.288 | 3 | 3 | 16.1 |
| Protein phosphatase 2A, catalytic subunit, β1 | LQEVPHEGPMCDLLWSDPDDR(640) QITQVYGFYDECLR(799) SPDTNYLFMGDYVDR(897) | *-* | 3.96E-49 | 162.65(640)  249.32(799)  344.54(897) | 258 | 29.646 | 3 | 3 | 19.4 |
| pyruvate kinase 3 | AEVSDVGNAIMDGADCVMLSGETAK(20) GDYPLVCVR(324) MGVDMVFASFIR(706) NIDSIIEEGDGIMIAR(737) | *Litopenaeus vannamei* | 5.74E-40 | 200.74(20)  133.23(324)  155.97(706)  312.49(737) | 229 | 24.774 | 4 | 4 | 27.1 |
| GDP mannose-4,6-dehydratase | ALVTDMMQADIDLMR(63) EIYWEGSGVDEVAK(211) | *Tribolium castaneum* | 1.48E-32 | 284.24(63)  280.21(211) | 94 | 10.805 | 2 | 2 | 30.9 |
| Isocitrate dehydrogenase | TSTNPVASIFAWTR | *Tribolium castaneum* | 0.00072306 | 168.89 | 168 | 18.971 | 1 | 1 | 8.3 |
| Inorganic pyrophosphatase | VDLWHYVALK(1014) VIMETHESWQHLVEGK(1042) | *Harpegnathos saltator* | 5.55E-09 | 208.22(1014)  210.64(1042) | 100 | 11.138 | 2 | 2 | 26 |
| AICAR transformylase | ANDAIITPDLLSNVVTK(69) TIFGLTLEQR(951) | *Glossina morsitans morsitans* | 1.48E-06 | 183.23(69)  180.67(951) | 313 | 34.151 | 2 | 2 | 8.6 |
| cytoplasmic manganese superoxide dismutase | GAHALPPLQYDYGALEPHICTTIMQIHHTK | *Xantho poressa* | 9.12E-13 | 167.59 | 66 | 7.3814 | 1 | 1 | 45.5 |
| cystathionine-beta-synthase | EGYDQLPVVDQEGMIR(199) VSTLHLPAPLTVLPTIACQDAIAIMQR(1091) | *Xenopus (Silurana) tropicalis* | 1.63E-10 | 165.86(199)  157.88(1091) | 386 | 42.657 | 2 | 2 | 11.1 |
| aspartyl/asparaginyl beta-hydroxylase | FFFHLGDALQR | *Culex quinquefasciatus* | 0.012559 | 130.66 | 37 | 4.1926 | 1 | 1 | 29.7 |
| aldehyde dehydrogenase 7 family, member A1 | HLLELGGNNAIIVDEDADPEMVVR(407) LILHHTLYDTILER(586) | *Tribolium castaneum* | 4.56E-15 | 179.95(407)  221.55(586) | 396 | 42.403 | 2 | 2 | 9.6 |
| **Glycolytic enzymes** |  |  |  |  |  |  |  |  |  |
| glutamate dehydrogenase | ESNYHLLESVQESLER | *Litopenaeus vannamei* | 0.0021338 | 161.21 | 315 | 34.486 | 1 | 1 | 5.1 |
| alpha glucosidase 2 | REPWLFDAETLR(833) WYQAAAFQPFMR(1123) | *Aedes aegypt*i | 7.78E-08 | 148.18(833)  202.56(1123) | 676 | 77.433 | 2 | 2 | 3.6 |
| aspartate aminotransferase | IVAAILTNPELR | *Aedes aegypti* | 0.00024251 | 175.86 | 351 | 38.843 | 1 | 1 | 3.4 |
| transaldolase | ALGGCDLLTIGPK(50) FLEELQNSTEPVVQHLSEASAK(274) ILDWYVANTDQK(471) | *Aedes aegypti* | 1.58E-26 | 201.17(50)  176.81(274)  275.76(471) | 298 | 33.553 | 3 | 3 | 15.8 |
| fructose 1,6-bisphosphatase | - | *Marsupenaeus japonicus* | 1.33E-81 |  | 313 | 33.795 | 8 | 8 | 33.5 |
| fructose-6-phosphate aminotransferase | - |  | 1.09E-19 |  | 285 | 31.284 | 4 | 4 | 16.5 |
| UDP-glucose 4-epimerase | - |  | 1.34E-07 |  | 85 | 9.7071 | 3 | 3 | 48.2 |
| acid ceramidase isoform c | LLAPVYFILGGTQPGEGCIITR | *Homo sapiens* | 2.09E-07 | 199.08 | 120 | 13.59 | 1 | 1 | 18.3 |
| fibrillarin protein | MVCGMVDTIFADVAQPDQAR | *Glossina morsitans morsitans* | 0.0059667 | 118.21 | 151 | 16.589 | 1 | 1 | 13.2 |
| **Crustacean molting protein** | |  |  |  |  |  |  |  |  |
| cryptocyanin | - | *Metacarcinus magister* | 4.85E-109 |  | 653 | 75.767 | 13 | 4 | 15.8 |
| cryptocyanin 2 | - | *Metacarcinus magister* | 4.57E-127 |  | 674 | 78.26 | 13 | 3 | 14.8 |
| hemocyanin subunit 1 | - |  | 2.81E-29 |  | 662 | 76.277 | 6 | 0 | 5.6 |
| beta-1,3-D-glucan binding protein | - | *Pacifastacus leniusculus* | 8.53E-42 |  | 271 | 30.24 | 8 | 8 | 33.6 |
| Iron/zinc purple acid phosphatase-like protein | QYEWLIEDLEQATQPEVR | *Camponotus floridanus* | 3.41E-12 | 243.9 | 228 | 26.693 | 1 | 1 | 7.9 |
| similar to past-1 | IGPEPTTDGFIAVMYGDTER(457) IILLFDAHK(461) VYIGSYWNEPLR(1110) | *Nasonia vitripennis* | 4.83E-16 | 226.74(457)  123.69(461)  199.03(1110) | 227 | 25.796 | 3 | 3 | 18.1 |
| calponin | ATYMHPEWPGPWLGPR | *Chironomus riparius* | 0.00011439 | 208.27 | 76 | 8.169 | 1 | 1 | 21.1 |
| lectin | PLWNHILFTYGK | *Eriocheir sinensis* | 0.0020063 | 148.56 | 160 | 17.834 | 1 | 1 | 7.5 |
| ferritin 3 | AGISGLGEFLFDK(31) AGISGLGEFLFDKEFE(32) IVLQAIAAPPQQEWGNCNDALQAALDLEK(511) | *Eriocheir sinensis* | 1.96E-08 | 164.81(31)  203.32(32)  74.222(511) | 170 | 19.573 | 3 | 3 | 26.5 |
| alpha 2-macroglobulin | - | *Eriocheir sinensis* | 3.50E-82 |  | 1457 | 162.1 | 13 | 10 | 12.8 |
| laminin receptor | FTPGAFTNQIQAAFR | *Litopenaeus vannamei* | 1.57E-10 | 242.8 | 71 | 7.7207 | 1 | 1 | 21.1 |
| vitellogenin | SLLTNILIPDNFER | *Eriocheir sinensis* | 0.0019762 | 153.81 | 159 | 18.121 | 1 | 1 | 8.8 |
| profilin/chicadae | SWQHYVDQQLMGSGVVSK | *Litopenaeus vannamei* | 8.68E-07 | 216.26 | 37 | 4.0585 | 1 | 1 | 48.6 |
| peroxiredoxin | - | *Penaeus monodon* | 2.55E-21 |  | 235 | 26.241 | 4 | 4 | 23 |
| Cct2 protein | SLHDALCVLTQTVK | *Danio rerio* | 0.0022485 | 141.58 | 128 | 13.631 | 1 | 1 | 10.9 |
| **Signal transducers** |  |  |  |  |  |  |  |  |  |
| Ras-related protein Rab-1A | GAHGIIVVYDTTDQESFNNVK | *Scylla paramamosain* | 1.31E-103 | 220.27 | 132 | 14.501 | 3 | 1 | 40.9 |
| Protein kinase C-binding protein 1 | RQMEAQMMEGR | *Harpegnathos saltator* | 0.14495 | 83.869 | 103 | 12.359 | 1 | 1 | 10.7 |
| GTP-binding protein SAR1b | AELDSLLTDDQLSNVPIVILGNK | *Caligus rogercresseyi* | 7.33E-13 | 240.13 | 121 | 13.652 | 1 | 1 | 19 |
| Eukaryotic translation initiation factor 3 subunit E Short=eIF3e | HLVFPLMEFLSVK | *Eriocheir sinensis* | 0.01511 | 124.67 | 172 | 20.06 | 1 | 1 | 7.6 |
| 14-3-3-like protein | EICQDVLGLLDK | *Penaeus monodon* | 2.23E-17 | 292.06 | 69 | 7.8549 | 1 | 1 | 17.4 |
| eukaryotic initiation factor 4A | - | *Callinectes sapidus* | 7.24E-43 |  | 432 | 48.725 | 8 | 8 | 26.2 |
| Eukaryotic translation initiation factor 2A | NTVSVNPVGNIVLVGGFGNISPR | *Tribolium castaneum* | 3.87E-07 | 171.13 | 313 | 34.443 | 1 | 1 | 7.3 |
| ADP ribosylation factor 4 | DAVLLVFANK(117) LGLNQLR(573) QDLPNAMTAAELTDR(787) | *Marsupenaeus japonicus* | 1.61E-22 | 193.82(117)  154.11(573)  271.64(787) | 70 | 8.002 | 3 | 3 | 45.7 |
| Eukaryotic peptide chain release factor GTP-binding subunit ERF3A | IFDAQVVILEHK | *Harpegnathos saltator* | 0.0064322 | 134.3 | 130 | 14.4 | 1 | 1 | 9.2 |
| eukaryotic translation initiation factor 3 subunit H | LGFESLFQEIR | *Bombyx mori* | 5.64E-05 | 192.38 | 270 | 31.219 | 1 | 1 | 4.1 |
| bifunctional aminoacyl-tRNA synthetase | VYPTYDFACPIVDSVEGVTHALR | *Gallus gallus* | 0.02418 | 104.88 | 99 | 11.691 | 1 | 1 | 23.2 |
| receptor for activated protein kinase c1 | LWDLAAGK(683) NFPDMILSASR(735) | *Penaeus monodon* | 0.00013624 | 108.46(683)  162.26(735) | 99 | 11.305 | 2 | 2 | 19.2 |
| muscle elongation factor 1 gamma | DPLDAFPAGNFNMDDFKR  (159)  IFMSCNLIGGMFQR  (446) | *Procambarus clarkii* | 6.97E-09 | 148.03(159)  223.22(446) | 173 | 20.19 | 2 | 2 | 18.5 |
| UMP-CMP kinase 1-like | SADEIFSDVEK | *Saccoglossus kowalevskii* | 0.021424 | 121.9 | 55 | 6.3 | 1 | 1 | 20 |
| similar to Chain D, Crystal Structure Of Arp23 COMPLEX | DNTINLIHIFR | *Gallus gallus* | 0.0005483 | 183.03 | 142 | 16.505 | 1 | 1 | 7.7 |
| DEAD box ATP-dependent RNA helicase | ILVATNLFGR | *Tribolium castaneum* | 0.00056295 | 175.93 | 142 | 16.396 | 1 | 1 | 7 |
| **Transporters and protein trafficking** | |  |  |  |  |  |  |  |  |
| Basement membrane-specific heparan sulfate proteoglycan core | SVDLPTLLIVTGVVPR | *Harpegnathos saltator* | 1.06E-06 | 221.86 | 262 | 28.319 | 1 | 1 | 6.1 |
| importin beta-3 | FEPYLPLVMGPVLK(254) LVLEQVVTTIASVADTAEEK(677) | *Culex quinquefasciatus* | 0.00010066 | 174.05(254)  83.894(677) | 326 | 36.193 | 2 | 2 | 10.4 |
| beta-1,3-glucuronyltransferase | VSMFPVGLVTK | *Tribolium castaneum* | 0.0011252 | 174.41 | 255 | 29.372 | 1 | 1 | 4.3 |
| Eukaryotic peptide chain release factor GTP-binding subunit ERF3A | IFDAQVVILEHK | *Harpegnathos saltator* | 0.0064322 | 134.3 | 130 | 14.4 | 1 | 1 | 9.2 |
| coatomer subunit alpha | SAGLTSVWVAR(847) SQLALDHVLAGAFESATR(904) TLELPVYVTR(960) | *Gallus gallus* | 8.19E-13 | 182.64(847)  221.59(904)  138.08(960) | 814 | 90.21 | 3 | 3 | 4.8 |
| alpha actinin CG4376-PB | ENVDSDTAEQVIDSFR | *Tribolium castaneum* | 3.79E-07 | 235.22 | 298 | 34.204 | 1 | 1 | 5.4 |
| coatomer protein complex, subunit beta 2 | TLYLLGYIPK | *Taeniopygia guttata* | 0.0079845 | 150.08 | 246 | 27.914 | 1 | 1 | 4.1 |
| Coatomer subunit beta | GDLQMAQTCLLR | *Harpegnathos saltator* | 1.02E-07 | 231.17 | 158 | 17.501 | 1 | 1 | 7.6 |
| coatomer gamma subunit | YILNPPQFALPSLER | *Loa loa* | 0.00023394 | 203.7 | 64 | 7.3785 | 1 | 1 | 23.4 |
| **Defense molecules** |  |  |  |  |  |  |  |  |  |
| Catalase | LVNNIAGHLINAQEFLQDR | *Scylla paramamosain* | 2.28E-20 | 294 | 192 | 21.673 | 1 | 1 | 9.9 |
| polyubiquitin | TITLEVEPSDTIENVK | *Eriocheir sinensis* | 1.14E-06 | 220.4 | 305 | 34.336 | 1 | 1 | 21 |
| ubiquitin carboxyl-terminal esterase L3 | AHEESAQEGQTEAPDR(38) EAQVNEHFVAFVHVDGK(179) QFVGNACGTVALIHAIANNR(792) | *Scylla paramamosain* | 1.81E-10 | 119.96(38)  138.21(179)  197.75(792) | 126 | 14.131 | 3 | 3 | 42.1 |
| ubiquitin-protein ligase |  |  | 0.0061816 |  | 98 | 11.415 | 1 | 1 | 11.2 |
| proteasome (prosome, macropain) 26S subunit, non-ATPase, 1 | SPEQIPSMVSLLSESYNPHVR | *Xenopus tropicalis* | 5.40E-06 | 183.74 | 317 | 34.145 | 1 | 1 | 6.6 |
| proteasome 26S non-ATPase subunit 2-like | NECDPALALLSDYISHR | *Saccoglossus kowalevskii* | 0.0034412 | 141.85 | 214 | 22.892 | 1 | 1 | 7.9 |
| 20S proteasome alpha subunit | ITSPLMIPSTIEK(506)LFQVEYAIEAIK(559) | *Scylla paramamosain* | 5.81E-09 | 160.67(506)  201(559) | 103 | 11.366 | 2 | 2 | 24.3 |
| **Chaperone molecules** | |  |  |  |  |  |  |  |  |
| heat shock protein 70 kDa | FDLTGIPPAPR(250) TTPSYVAFTDTER(987) | *Cyanagraea praedator* | 0.00027668 | 133.81(250)  111.52(987) | 635 | 69.746 | 2 | 1 | 3.8 |
| glucose-regulated protein 78 | - | *Fenneropenaeus chinensis* | 3.26E-54 |  | 352 | 39.331 | 7 | 4 | 28.7 |
| heat shock protein 90-1 | ALLFLPR(56) RAPFDLFENR(831) | *Portunus trituberculatus* | 0.00063531 | 137.5(56)  130.41(831) | 721 | 83.345 | 2 | 2 | 2.4 |
| glucose-regulated protein 94 | LLALTDKEELTTNPEMVIR | *Crassostrea gigas* | 4.45E-07 | 219.52 | 97 | 11.227 | 1 | 1 | 19.6 |
| cyclophilin A | HVVFGSVVEGMDVVR | *Scylla paramamosain* | 0.0004638 | 191.05 | 80 | 8.4083 | 1 | 1 | 18.8 |

Note：PEP (Posterior error probability) is the probability of an individual match.
