# Supplementary material for: A Catalog of Proteins Expressed in the AG Secreted Fluid during the Mature Phase of the Chinese Mitten Crabs (Eriocheir sinensis)
Source: PLoS One. 2015 Aug 25;10(8):e0136266. doi: 10.1371/journal.pone.0136266 (PMC4549300; doi:10.1371/journal.pone.0136266)
Supplement: S4 Table — The Enzyme Commission (EC) number of the best match protein (E-value < e-15) was accepted and exported. (DOC) [file pone.0136266.s004.doc]

| **Table S3 Metabolic pathways by KEGG analysis involved in ASG protomics from *Eriocheir sinensis*** | | | |
| --- | --- | --- | --- |
| **No.** | **Pathway** | **Proteins with pathway annotation (394)** | **Pathway ID** |
| 1 | [Metabolic pathways](../../../../H:%5CP%E8%9B%8B%E7%99%BD%E8%B4%A8%E7%BB%84%E5%AD%A6%E6%96%87%E7%AB%A0%5C20120515%E5%89%AF%E6%80%A7%E8%85%BA%E5%92%8C%E7%B2%BE%E5%B7%A2%E8%9B%8B%E7%99%BD%E8%B4%A8%E7%BB%84%E6%9C%80%E6%96%B0%E5%88%86%E6%9E%90%5C20120221_rmx%5Cannotation%5Crmx2_kegg%5Crmx2.htm#gene1) | 125 (31.73%) | ko01100 |
| 2 | [Pathogenic Escherichia coli infection](../../../../H:%5CP%E8%9B%8B%E7%99%BD%E8%B4%A8%E7%BB%84%E5%AD%A6%E6%96%87%E7%AB%A0%5C20120515%E5%89%AF%E6%80%A7%E8%85%BA%E5%92%8C%E7%B2%BE%E5%B7%A2%E8%9B%8B%E7%99%BD%E8%B4%A8%E7%BB%84%E6%9C%80%E6%96%B0%E5%88%86%E6%9E%90%5C20120221_rmx%5Cannotation%5Crmx2_kegg%5Crmx2.htm#gene2) | 28 (7.11%) | ko05130 |
| 3 | [Phagosome](../../../../H:%5CP%E8%9B%8B%E7%99%BD%E8%B4%A8%E7%BB%84%E5%AD%A6%E6%96%87%E7%AB%A0%5C20120515%E5%89%AF%E6%80%A7%E8%85%BA%E5%92%8C%E7%B2%BE%E5%B7%A2%E8%9B%8B%E7%99%BD%E8%B4%A8%E7%BB%84%E6%9C%80%E6%96%B0%E5%88%86%E6%9E%90%5C20120221_rmx%5Cannotation%5Crmx2_kegg%5Crmx2.htm#gene3) | 25 (6.35%) | ko04145 |
| 4 | [Proteasome](../../../../H:%5CP%E8%9B%8B%E7%99%BD%E8%B4%A8%E7%BB%84%E5%AD%A6%E6%96%87%E7%AB%A0%5C20120515%E5%89%AF%E6%80%A7%E8%85%BA%E5%92%8C%E7%B2%BE%E5%B7%A2%E8%9B%8B%E7%99%BD%E8%B4%A8%E7%BB%84%E6%9C%80%E6%96%B0%E5%88%86%E6%9E%90%5C20120221_rmx%5Cannotation%5Crmx2_kegg%5Crmx2.htm#gene4) | 24 (6.09%) | ko03050 |
| 5 | [Tyrosine metabolism](../../../../H:%5CP%E8%9B%8B%E7%99%BD%E8%B4%A8%E7%BB%84%E5%AD%A6%E6%96%87%E7%AB%A0%5C20120515%E5%89%AF%E6%80%A7%E8%85%BA%E5%92%8C%E7%B2%BE%E5%B7%A2%E8%9B%8B%E7%99%BD%E8%B4%A8%E7%BB%84%E6%9C%80%E6%96%B0%E5%88%86%E6%9E%90%5C20120221_rmx%5Cannotation%5Crmx2_kegg%5Crmx2.htm#gene5) | 22 (5.58%) | ko00350 |
| 6 | [Protein processing in endoplasmic reticulum](../../../../H:%5CP%E8%9B%8B%E7%99%BD%E8%B4%A8%E7%BB%84%E5%AD%A6%E6%96%87%E7%AB%A0%5C20120515%E5%89%AF%E6%80%A7%E8%85%BA%E5%92%8C%E7%B2%BE%E5%B7%A2%E8%9B%8B%E7%99%BD%E8%B4%A8%E7%BB%84%E6%9C%80%E6%96%B0%E5%88%86%E6%9E%90%5C20120221_rmx%5Cannotation%5Crmx2_kegg%5Crmx2.htm#gene6) | 21 (5.33%) | ko04141 |
| 7 | [Amino sugar and nucleotide sugar metabolism](../../../../H:%5CP%E8%9B%8B%E7%99%BD%E8%B4%A8%E7%BB%84%E5%AD%A6%E6%96%87%E7%AB%A0%5C20120515%E5%89%AF%E6%80%A7%E8%85%BA%E5%92%8C%E7%B2%BE%E5%B7%A2%E8%9B%8B%E7%99%BD%E8%B4%A8%E7%BB%84%E6%9C%80%E6%96%B0%E5%88%86%E6%9E%90%5C20120221_rmx%5Cannotation%5Crmx2_kegg%5Crmx2.htm#gene7) | 20 (5.08%) | ko00520 |
| 8 | [Ribosome](../../../../H:%5CP%E8%9B%8B%E7%99%BD%E8%B4%A8%E7%BB%84%E5%AD%A6%E6%96%87%E7%AB%A0%5C20120515%E5%89%AF%E6%80%A7%E8%85%BA%E5%92%8C%E7%B2%BE%E5%B7%A2%E8%9B%8B%E7%99%BD%E8%B4%A8%E7%BB%84%E6%9C%80%E6%96%B0%E5%88%86%E6%9E%90%5C20120221_rmx%5Cannotation%5Crmx2_kegg%5Crmx2.htm#gene8) | 19 (4.82%) | ko03010 |
| 9 | [Lysosome](../../../../H:%5CP%E8%9B%8B%E7%99%BD%E8%B4%A8%E7%BB%84%E5%AD%A6%E6%96%87%E7%AB%A0%5C20120515%E5%89%AF%E6%80%A7%E8%85%BA%E5%92%8C%E7%B2%BE%E5%B7%A2%E8%9B%8B%E7%99%BD%E8%B4%A8%E7%BB%84%E6%9C%80%E6%96%B0%E5%88%86%E6%9E%90%5C20120221_rmx%5Cannotation%5Crmx2_kegg%5Crmx2.htm#gene9) | 18 (4.57%) | ko04142 |
| 10 | [Gap junction](../../../../H:%5CP%E8%9B%8B%E7%99%BD%E8%B4%A8%E7%BB%84%E5%AD%A6%E6%96%87%E7%AB%A0%5C20120515%E5%89%AF%E6%80%A7%E8%85%BA%E5%92%8C%E7%B2%BE%E5%B7%A2%E8%9B%8B%E7%99%BD%E8%B4%A8%E7%BB%84%E6%9C%80%E6%96%B0%E5%88%86%E6%9E%90%5C20120221_rmx%5Cannotation%5Crmx2_kegg%5Crmx2.htm#gene10) | 17 (4.31%) | ko04540 |
| 11 | [Glycolysis / Gluconeogenesis](../../../../H:%5CP%E8%9B%8B%E7%99%BD%E8%B4%A8%E7%BB%84%E5%AD%A6%E6%96%87%E7%AB%A0%5C20120515%E5%89%AF%E6%80%A7%E8%85%BA%E5%92%8C%E7%B2%BE%E5%B7%A2%E8%9B%8B%E7%99%BD%E8%B4%A8%E7%BB%84%E6%9C%80%E6%96%B0%E5%88%86%E6%9E%90%5C20120221_rmx%5Cannotation%5Crmx2_kegg%5Crmx2.htm#gene11) | 17 (4.31%) | ko00010 |
| 12 | [Regulation of actin cytoskeleton](../../../../H:%5CP%E8%9B%8B%E7%99%BD%E8%B4%A8%E7%BB%84%E5%AD%A6%E6%96%87%E7%AB%A0%5C20120515%E5%89%AF%E6%80%A7%E8%85%BA%E5%92%8C%E7%B2%BE%E5%B7%A2%E8%9B%8B%E7%99%BD%E8%B4%A8%E7%BB%84%E6%9C%80%E6%96%B0%E5%88%86%E6%9E%90%5C20120221_rmx%5Cannotation%5Crmx2_kegg%5Crmx2.htm#gene12) | 15 (3.81%) | ko04810 |
| 13 | [RNA transport](../../../../H:%5CP%E8%9B%8B%E7%99%BD%E8%B4%A8%E7%BB%84%E5%AD%A6%E6%96%87%E7%AB%A0%5C20120515%E5%89%AF%E6%80%A7%E8%85%BA%E5%92%8C%E7%B2%BE%E5%B7%A2%E8%9B%8B%E7%99%BD%E8%B4%A8%E7%BB%84%E6%9C%80%E6%96%B0%E5%88%86%E6%9E%90%5C20120221_rmx%5Cannotation%5Crmx2_kegg%5Crmx2.htm#gene13) | 15 (3.81%) | ko03013 |
| 14 | [Aminoacyl-tRNA biosynthesis](../../../../H:%5CP%E8%9B%8B%E7%99%BD%E8%B4%A8%E7%BB%84%E5%AD%A6%E6%96%87%E7%AB%A0%5C20120515%E5%89%AF%E6%80%A7%E8%85%BA%E5%92%8C%E7%B2%BE%E5%B7%A2%E8%9B%8B%E7%99%BD%E8%B4%A8%E7%BB%84%E6%9C%80%E6%96%B0%E5%88%86%E6%9E%90%5C20120221_rmx%5Cannotation%5Crmx2_kegg%5Crmx2.htm#gene14) | 15 (3.81%) | ko00970 |
| 15 | [Melanogenesis](../../../../H:%5CP%E8%9B%8B%E7%99%BD%E8%B4%A8%E7%BB%84%E5%AD%A6%E6%96%87%E7%AB%A0%5C20120515%E5%89%AF%E6%80%A7%E8%85%BA%E5%92%8C%E7%B2%BE%E5%B7%A2%E8%9B%8B%E7%99%BD%E8%B4%A8%E7%BB%84%E6%9C%80%E6%96%B0%E5%88%86%E6%9E%90%5C20120221_rmx%5Cannotation%5Crmx2_kegg%5Crmx2.htm#gene15) | 14 (3.55%) | ko04916 |
| 16 | [Riboflavin metabolism](../../../../H:%5CP%E8%9B%8B%E7%99%BD%E8%B4%A8%E7%BB%84%E5%AD%A6%E6%96%87%E7%AB%A0%5C20120515%E5%89%AF%E6%80%A7%E8%85%BA%E5%92%8C%E7%B2%BE%E5%B7%A2%E8%9B%8B%E7%99%BD%E8%B4%A8%E7%BB%84%E6%9C%80%E6%96%B0%E5%88%86%E6%9E%90%5C20120221_rmx%5Cannotation%5Crmx2_kegg%5Crmx2.htm#gene16) | 14 (3.55%) | ko00740 |
| 17 | [Pyruvate metabolism](../../../../H:%5CP%E8%9B%8B%E7%99%BD%E8%B4%A8%E7%BB%84%E5%AD%A6%E6%96%87%E7%AB%A0%5C20120515%E5%89%AF%E6%80%A7%E8%85%BA%E5%92%8C%E7%B2%BE%E5%B7%A2%E8%9B%8B%E7%99%BD%E8%B4%A8%E7%BB%84%E6%9C%80%E6%96%B0%E5%88%86%E6%9E%90%5C20120221_rmx%5Cannotation%5Crmx2_kegg%5Crmx2.htm#gene17) | 13 (3.3%) | ko00620 |
| 18 | [Purine metabolism](../../../../H:%5CP%E8%9B%8B%E7%99%BD%E8%B4%A8%E7%BB%84%E5%AD%A6%E6%96%87%E7%AB%A0%5C20120515%E5%89%AF%E6%80%A7%E8%85%BA%E5%92%8C%E7%B2%BE%E5%B7%A2%E8%9B%8B%E7%99%BD%E8%B4%A8%E7%BB%84%E6%9C%80%E6%96%B0%E5%88%86%E6%9E%90%5C20120221_rmx%5Cannotation%5Crmx2_kegg%5Crmx2.htm#gene18) | 12 (3.05%) | ko00230 |
| 19 | [Citrate cycle (TCA cycle)](../../../../H:%5CP%E8%9B%8B%E7%99%BD%E8%B4%A8%E7%BB%84%E5%AD%A6%E6%96%87%E7%AB%A0%5C20120515%E5%89%AF%E6%80%A7%E8%85%BA%E5%92%8C%E7%B2%BE%E5%B7%A2%E8%9B%8B%E7%99%BD%E8%B4%A8%E7%BB%84%E6%9C%80%E6%96%B0%E5%88%86%E6%9E%90%5C20120221_rmx%5Cannotation%5Crmx2_kegg%5Crmx2.htm#gene19) | 11 (2.79%) | ko00020 |
| 20 | [Amoebiasis](../../../../H:%5CP%E8%9B%8B%E7%99%BD%E8%B4%A8%E7%BB%84%E5%AD%A6%E6%96%87%E7%AB%A0%5C20120515%E5%89%AF%E6%80%A7%E8%85%BA%E5%92%8C%E7%B2%BE%E5%B7%A2%E8%9B%8B%E7%99%BD%E8%B4%A8%E7%BB%84%E6%9C%80%E6%96%B0%E5%88%86%E6%9E%90%5C20120221_rmx%5Cannotation%5Crmx2_kegg%5Crmx2.htm#gene20) | 10 (2.54%) | ko05146 |
| 21 | [Galactose metabolism](../../../../H:%5CP%E8%9B%8B%E7%99%BD%E8%B4%A8%E7%BB%84%E5%AD%A6%E6%96%87%E7%AB%A0%5C20120515%E5%89%AF%E6%80%A7%E8%85%BA%E5%92%8C%E7%B2%BE%E5%B7%A2%E8%9B%8B%E7%99%BD%E8%B4%A8%E7%BB%84%E6%9C%80%E6%96%B0%E5%88%86%E6%9E%90%5C20120221_rmx%5Cannotation%5Crmx2_kegg%5Crmx2.htm#gene21) | 10 (2.54%) | ko00052 |
| 22 | [Glyoxylate and dicarboxylate metabolism](../../../../H:%5CP%E8%9B%8B%E7%99%BD%E8%B4%A8%E7%BB%84%E5%AD%A6%E6%96%87%E7%AB%A0%5C20120515%E5%89%AF%E6%80%A7%E8%85%BA%E5%92%8C%E7%B2%BE%E5%B7%A2%E8%9B%8B%E7%99%BD%E8%B4%A8%E7%BB%84%E6%9C%80%E6%96%B0%E5%88%86%E6%9E%90%5C20120221_rmx%5Cannotation%5Crmx2_kegg%5Crmx2.htm#gene22) | 10 (2.54%) | ko00630 |
| 23 | [Alanine, aspartate and glutamate metabolism](../../../../H:%5CP%E8%9B%8B%E7%99%BD%E8%B4%A8%E7%BB%84%E5%AD%A6%E6%96%87%E7%AB%A0%5C20120515%E5%89%AF%E6%80%A7%E8%85%BA%E5%92%8C%E7%B2%BE%E5%B7%A2%E8%9B%8B%E7%99%BD%E8%B4%A8%E7%BB%84%E6%9C%80%E6%96%B0%E5%88%86%E6%9E%90%5C20120221_rmx%5Cannotation%5Crmx2_kegg%5Crmx2.htm#gene23) | 10 (2.54%) | ko00250 |
| 24 | [Arginine and proline metabolism](../../../../H:%5CP%E8%9B%8B%E7%99%BD%E8%B4%A8%E7%BB%84%E5%AD%A6%E6%96%87%E7%AB%A0%5C20120515%E5%89%AF%E6%80%A7%E8%85%BA%E5%92%8C%E7%B2%BE%E5%B7%A2%E8%9B%8B%E7%99%BD%E8%B4%A8%E7%BB%84%E6%9C%80%E6%96%B0%E5%88%86%E6%9E%90%5C20120221_rmx%5Cannotation%5Crmx2_kegg%5Crmx2.htm#gene24) | 10 (2.54%) | ko00330 |
| 25 | [Peroxisome](../../../../H:%5CP%E8%9B%8B%E7%99%BD%E8%B4%A8%E7%BB%84%E5%AD%A6%E6%96%87%E7%AB%A0%5C20120515%E5%89%AF%E6%80%A7%E8%85%BA%E5%92%8C%E7%B2%BE%E5%B7%A2%E8%9B%8B%E7%99%BD%E8%B4%A8%E7%BB%84%E6%9C%80%E6%96%B0%E5%88%86%E6%9E%90%5C20120221_rmx%5Cannotation%5Crmx2_kegg%5Crmx2.htm#gene25) | 9 (2.28%) | ko04146 |
| 26 | [Spliceosome](../../../../H:%5CP%E8%9B%8B%E7%99%BD%E8%B4%A8%E7%BB%84%E5%AD%A6%E6%96%87%E7%AB%A0%5C20120515%E5%89%AF%E6%80%A7%E8%85%BA%E5%92%8C%E7%B2%BE%E5%B7%A2%E8%9B%8B%E7%99%BD%E8%B4%A8%E7%BB%84%E6%9C%80%E6%96%B0%E5%88%86%E6%9E%90%5C20120221_rmx%5Cannotation%5Crmx2_kegg%5Crmx2.htm#gene26) | 9 (2.28%) | ko03040 |
| 27 | [Glutathione metabolism](../../../../H:%5CP%E8%9B%8B%E7%99%BD%E8%B4%A8%E7%BB%84%E5%AD%A6%E6%96%87%E7%AB%A0%5C20120515%E5%89%AF%E6%80%A7%E8%85%BA%E5%92%8C%E7%B2%BE%E5%B7%A2%E8%9B%8B%E7%99%BD%E8%B4%A8%E7%BB%84%E6%9C%80%E6%96%B0%E5%88%86%E6%9E%90%5C20120221_rmx%5Cannotation%5Crmx2_kegg%5Crmx2.htm#gene27) | 9 (2.28%) | ko00480 |
| 28 | [Focal adhesion](../../../../H:%5CP%E8%9B%8B%E7%99%BD%E8%B4%A8%E7%BB%84%E5%AD%A6%E6%96%87%E7%AB%A0%5C20120515%E5%89%AF%E6%80%A7%E8%85%BA%E5%92%8C%E7%B2%BE%E5%B7%A2%E8%9B%8B%E7%99%BD%E8%B4%A8%E7%BB%84%E6%9C%80%E6%96%B0%E5%88%86%E6%9E%90%5C20120221_rmx%5Cannotation%5Crmx2_kegg%5Crmx2.htm#gene28) | 9 (2.28%) | ko04510 |
| 29 | [Pathways in cancer](../../../../H:%5CP%E8%9B%8B%E7%99%BD%E8%B4%A8%E7%BB%84%E5%AD%A6%E6%96%87%E7%AB%A0%5C20120515%E5%89%AF%E6%80%A7%E8%85%BA%E5%92%8C%E7%B2%BE%E5%B7%A2%E8%9B%8B%E7%99%BD%E8%B4%A8%E7%BB%84%E6%9C%80%E6%96%B0%E5%88%86%E6%9E%90%5C20120221_rmx%5Cannotation%5Crmx2_kegg%5Crmx2.htm#gene29) | 9 (2.28%) | ko05200 |
| 30 | [Pancreatic secretion](../../../../H:%5CP%E8%9B%8B%E7%99%BD%E8%B4%A8%E7%BB%84%E5%AD%A6%E6%96%87%E7%AB%A0%5C20120515%E5%89%AF%E6%80%A7%E8%85%BA%E5%92%8C%E7%B2%BE%E5%B7%A2%E8%9B%8B%E7%99%BD%E8%B4%A8%E7%BB%84%E6%9C%80%E6%96%B0%E5%88%86%E6%9E%90%5C20120221_rmx%5Cannotation%5Crmx2_kegg%5Crmx2.htm#gene30) | 9 (2.28%) | ko04972 |
| 31 | [Antigen processing and presentation](../../../../H:%5CP%E8%9B%8B%E7%99%BD%E8%B4%A8%E7%BB%84%E5%AD%A6%E6%96%87%E7%AB%A0%5C20120515%E5%89%AF%E6%80%A7%E8%85%BA%E5%92%8C%E7%B2%BE%E5%B7%A2%E8%9B%8B%E7%99%BD%E8%B4%A8%E7%BB%84%E6%9C%80%E6%96%B0%E5%88%86%E6%9E%90%5C20120221_rmx%5Cannotation%5Crmx2_kegg%5Crmx2.htm#gene31) | 9 (2.28%) | ko04612 |
| 32 | [Cysteine and methionine metabolism](../../../../H:%5CP%E8%9B%8B%E7%99%BD%E8%B4%A8%E7%BB%84%E5%AD%A6%E6%96%87%E7%AB%A0%5C20120515%E5%89%AF%E6%80%A7%E8%85%BA%E5%92%8C%E7%B2%BE%E5%B7%A2%E8%9B%8B%E7%99%BD%E8%B4%A8%E7%BB%84%E6%9C%80%E6%96%B0%E5%88%86%E6%9E%90%5C20120221_rmx%5Cannotation%5Crmx2_kegg%5Crmx2.htm#gene32) | 8 (2.03%) | ko00270 |
| 33 | [Leukocyte transendothelial migration](../../../../H:%5CP%E8%9B%8B%E7%99%BD%E8%B4%A8%E7%BB%84%E5%AD%A6%E6%96%87%E7%AB%A0%5C20120515%E5%89%AF%E6%80%A7%E8%85%BA%E5%92%8C%E7%B2%BE%E5%B7%A2%E8%9B%8B%E7%99%BD%E8%B4%A8%E7%BB%84%E6%9C%80%E6%96%B0%E5%88%86%E6%9E%90%5C20120221_rmx%5Cannotation%5Crmx2_kegg%5Crmx2.htm#gene33) | 8 (2.03%) | ko04670 |
| 34 | [Protein digestion and absorption](../../../../H:%5CP%E8%9B%8B%E7%99%BD%E8%B4%A8%E7%BB%84%E5%AD%A6%E6%96%87%E7%AB%A0%5C20120515%E5%89%AF%E6%80%A7%E8%85%BA%E5%92%8C%E7%B2%BE%E5%B7%A2%E8%9B%8B%E7%99%BD%E8%B4%A8%E7%BB%84%E6%9C%80%E6%96%B0%E5%88%86%E6%9E%90%5C20120221_rmx%5Cannotation%5Crmx2_kegg%5Crmx2.htm#gene34) | 8 (2.03%) | ko04974 |
| 35 | [Shigellosis](../../../../H:%5CP%E8%9B%8B%E7%99%BD%E8%B4%A8%E7%BB%84%E5%AD%A6%E6%96%87%E7%AB%A0%5C20120515%E5%89%AF%E6%80%A7%E8%85%BA%E5%92%8C%E7%B2%BE%E5%B7%A2%E8%9B%8B%E7%99%BD%E8%B4%A8%E7%BB%84%E6%9C%80%E6%96%B0%E5%88%86%E6%9E%90%5C20120221_rmx%5Cannotation%5Crmx2_kegg%5Crmx2.htm#gene35) | 8 (2.03%) | ko05131 |
| 36 | [Tight junction](../../../../H:%5CP%E8%9B%8B%E7%99%BD%E8%B4%A8%E7%BB%84%E5%AD%A6%E6%96%87%E7%AB%A0%5C20120515%E5%89%AF%E6%80%A7%E8%85%BA%E5%92%8C%E7%B2%BE%E5%B7%A2%E8%9B%8B%E7%99%BD%E8%B4%A8%E7%BB%84%E6%9C%80%E6%96%B0%E5%88%86%E6%9E%90%5C20120221_rmx%5Cannotation%5Crmx2_kegg%5Crmx2.htm#gene36) | 8 (2.03%) | ko04530 |
| 37 | [Fructose and mannose metabolism](../../../../H:%5CP%E8%9B%8B%E7%99%BD%E8%B4%A8%E7%BB%84%E5%AD%A6%E6%96%87%E7%AB%A0%5C20120515%E5%89%AF%E6%80%A7%E8%85%BA%E5%92%8C%E7%B2%BE%E5%B7%A2%E8%9B%8B%E7%99%BD%E8%B4%A8%E7%BB%84%E6%9C%80%E6%96%B0%E5%88%86%E6%9E%90%5C20120221_rmx%5Cannotation%5Crmx2_kegg%5Crmx2.htm#gene37) | 8 (2.03%) | ko00051 |
| 38 | [Endocytosis](../../../../H:%5CP%E8%9B%8B%E7%99%BD%E8%B4%A8%E7%BB%84%E5%AD%A6%E6%96%87%E7%AB%A0%5C20120515%E5%89%AF%E6%80%A7%E8%85%BA%E5%92%8C%E7%B2%BE%E5%B7%A2%E8%9B%8B%E7%99%BD%E8%B4%A8%E7%BB%84%E6%9C%80%E6%96%B0%E5%88%86%E6%9E%90%5C20120221_rmx%5Cannotation%5Crmx2_kegg%5Crmx2.htm#gene38) | 7 (1.78%) | ko04144 |
| 39 | [Tryptophan metabolism](../../../../H:%5CP%E8%9B%8B%E7%99%BD%E8%B4%A8%E7%BB%84%E5%AD%A6%E6%96%87%E7%AB%A0%5C20120515%E5%89%AF%E6%80%A7%E8%85%BA%E5%92%8C%E7%B2%BE%E5%B7%A2%E8%9B%8B%E7%99%BD%E8%B4%A8%E7%BB%84%E6%9C%80%E6%96%B0%E5%88%86%E6%9E%90%5C20120221_rmx%5Cannotation%5Crmx2_kegg%5Crmx2.htm#gene39) | 7 (1.78%) | ko00380 |
| 40 | [Gastric acid secretion](../../../../H:%5CP%E8%9B%8B%E7%99%BD%E8%B4%A8%E7%BB%84%E5%AD%A6%E6%96%87%E7%AB%A0%5C20120515%E5%89%AF%E6%80%A7%E8%85%BA%E5%92%8C%E7%B2%BE%E5%B7%A2%E8%9B%8B%E7%99%BD%E8%B4%A8%E7%BB%84%E6%9C%80%E6%96%B0%E5%88%86%E6%9E%90%5C20120221_rmx%5Cannotation%5Crmx2_kegg%5Crmx2.htm#gene40) | 7 (1.78%) | ko04971 |
| 41 | [Pentose and glucuronate interconversions](../../../../H:%5CP%E8%9B%8B%E7%99%BD%E8%B4%A8%E7%BB%84%E5%AD%A6%E6%96%87%E7%AB%A0%5C20120515%E5%89%AF%E6%80%A7%E8%85%BA%E5%92%8C%E7%B2%BE%E5%B7%A2%E8%9B%8B%E7%99%BD%E8%B4%A8%E7%BB%84%E6%9C%80%E6%96%B0%E5%88%86%E6%9E%90%5C20120221_rmx%5Cannotation%5Crmx2_kegg%5Crmx2.htm#gene41) | 7 (1.78%) | ko00040 |
| 42 | [Pentose phosphate pathway](../../../../H:%5CP%E8%9B%8B%E7%99%BD%E8%B4%A8%E7%BB%84%E5%AD%A6%E6%96%87%E7%AB%A0%5C20120515%E5%89%AF%E6%80%A7%E8%85%BA%E5%92%8C%E7%B2%BE%E5%B7%A2%E8%9B%8B%E7%99%BD%E8%B4%A8%E7%BB%84%E6%9C%80%E6%96%B0%E5%88%86%E6%9E%90%5C20120221_rmx%5Cannotation%5Crmx2_kegg%5Crmx2.htm#gene42) | 7 (1.78%) | ko00030 |
| 43 | [Neurotrophin signaling pathway](../../../../H:%5CP%E8%9B%8B%E7%99%BD%E8%B4%A8%E7%BB%84%E5%AD%A6%E6%96%87%E7%AB%A0%5C20120515%E5%89%AF%E6%80%A7%E8%85%BA%E5%92%8C%E7%B2%BE%E5%B7%A2%E8%9B%8B%E7%99%BD%E8%B4%A8%E7%BB%84%E6%9C%80%E6%96%B0%E5%88%86%E6%9E%90%5C20120221_rmx%5Cannotation%5Crmx2_kegg%5Crmx2.htm#gene43) | 7 (1.78%) | ko04722 |
| 44 | [Adherens junction](../../../../H:%5CP%E8%9B%8B%E7%99%BD%E8%B4%A8%E7%BB%84%E5%AD%A6%E6%96%87%E7%AB%A0%5C20120515%E5%89%AF%E6%80%A7%E8%85%BA%E5%92%8C%E7%B2%BE%E5%B7%A2%E8%9B%8B%E7%99%BD%E8%B4%A8%E7%BB%84%E6%9C%80%E6%96%B0%E5%88%86%E6%9E%90%5C20120221_rmx%5Cannotation%5Crmx2_kegg%5Crmx2.htm#gene44) | 7 (1.78%) | ko04520 |
| 45 | [Porphyrin and chlorophyll metabolism](../../../../H:%5CP%E8%9B%8B%E7%99%BD%E8%B4%A8%E7%BB%84%E5%AD%A6%E6%96%87%E7%AB%A0%5C20120515%E5%89%AF%E6%80%A7%E8%85%BA%E5%92%8C%E7%B2%BE%E5%B7%A2%E8%9B%8B%E7%99%BD%E8%B4%A8%E7%BB%84%E6%9C%80%E6%96%B0%E5%88%86%E6%9E%90%5C20120221_rmx%5Cannotation%5Crmx2_kegg%5Crmx2.htm#gene45) | 6 (1.52%) | ko00860 |
| 46 | [Bacterial invasion of epithelial cells](../../../../H:%5CP%E8%9B%8B%E7%99%BD%E8%B4%A8%E7%BB%84%E5%AD%A6%E6%96%87%E7%AB%A0%5C20120515%E5%89%AF%E6%80%A7%E8%85%BA%E5%92%8C%E7%B2%BE%E5%B7%A2%E8%9B%8B%E7%99%BD%E8%B4%A8%E7%BB%84%E6%9C%80%E6%96%B0%E5%88%86%E6%9E%90%5C20120221_rmx%5Cannotation%5Crmx2_kegg%5Crmx2.htm#gene46) | 6 (1.52%) | ko05100 |
| 47 | [Propanoate metabolism](../../../../H:%5CP%E8%9B%8B%E7%99%BD%E8%B4%A8%E7%BB%84%E5%AD%A6%E6%96%87%E7%AB%A0%5C20120515%E5%89%AF%E6%80%A7%E8%85%BA%E5%92%8C%E7%B2%BE%E5%B7%A2%E8%9B%8B%E7%99%BD%E8%B4%A8%E7%BB%84%E6%9C%80%E6%96%B0%E5%88%86%E6%9E%90%5C20120221_rmx%5Cannotation%5Crmx2_kegg%5Crmx2.htm#gene47) | 6 (1.52%) | ko00640 |
| 48 | [Starch and sucrose metabolism](../../../../H:%5CP%E8%9B%8B%E7%99%BD%E8%B4%A8%E7%BB%84%E5%AD%A6%E6%96%87%E7%AB%A0%5C20120515%E5%89%AF%E6%80%A7%E8%85%BA%E5%92%8C%E7%B2%BE%E5%B7%A2%E8%9B%8B%E7%99%BD%E8%B4%A8%E7%BB%84%E6%9C%80%E6%96%B0%E5%88%86%E6%9E%90%5C20120221_rmx%5Cannotation%5Crmx2_kegg%5Crmx2.htm#gene48) | 6 (1.52%) | ko00500 |
| 49 | [Prostate cancer](../../../../H:%5CP%E8%9B%8B%E7%99%BD%E8%B4%A8%E7%BB%84%E5%AD%A6%E6%96%87%E7%AB%A0%5C20120515%E5%89%AF%E6%80%A7%E8%85%BA%E5%92%8C%E7%B2%BE%E5%B7%A2%E8%9B%8B%E7%99%BD%E8%B4%A8%E7%BB%84%E6%9C%80%E6%96%B0%E5%88%86%E6%9E%90%5C20120221_rmx%5Cannotation%5Crmx2_kegg%5Crmx2.htm#gene49) | 6 (1.52%) | ko05215 |
| 50 | [Viral myocarditis](../../../../H:%5CP%E8%9B%8B%E7%99%BD%E8%B4%A8%E7%BB%84%E5%AD%A6%E6%96%87%E7%AB%A0%5C20120515%E5%89%AF%E6%80%A7%E8%85%BA%E5%92%8C%E7%B2%BE%E5%B7%A2%E8%9B%8B%E7%99%BD%E8%B4%A8%E7%BB%84%E6%9C%80%E6%96%B0%E5%88%86%E6%9E%90%5C20120221_rmx%5Cannotation%5Crmx2_kegg%5Crmx2.htm#gene50) | 6 (1.52%) | ko05416 |
| 51 | [Dilated cardiomyopathy](../../../../H:%5CP%E8%9B%8B%E7%99%BD%E8%B4%A8%E7%BB%84%E5%AD%A6%E6%96%87%E7%AB%A0%5C20120515%E5%89%AF%E6%80%A7%E8%85%BA%E5%92%8C%E7%B2%BE%E5%B7%A2%E8%9B%8B%E7%99%BD%E8%B4%A8%E7%BB%84%E6%9C%80%E6%96%B0%E5%88%86%E6%9E%90%5C20120221_rmx%5Cannotation%5Crmx2_kegg%5Crmx2.htm#gene51) | 6 (1.52%) | ko05414 |
| 52 | [Complement and coagulation cascades](../../../../H:%5CP%E8%9B%8B%E7%99%BD%E8%B4%A8%E7%BB%84%E5%AD%A6%E6%96%87%E7%AB%A0%5C20120515%E5%89%AF%E6%80%A7%E8%85%BA%E5%92%8C%E7%B2%BE%E5%B7%A2%E8%9B%8B%E7%99%BD%E8%B4%A8%E7%BB%84%E6%9C%80%E6%96%B0%E5%88%86%E6%9E%90%5C20120221_rmx%5Cannotation%5Crmx2_kegg%5Crmx2.htm#gene52) | 6 (1.52%) | ko04610 |
| 53 | [Oocyte meiosis](../../../../H:%5CP%E8%9B%8B%E7%99%BD%E8%B4%A8%E7%BB%84%E5%AD%A6%E6%96%87%E7%AB%A0%5C20120515%E5%89%AF%E6%80%A7%E8%85%BA%E5%92%8C%E7%B2%BE%E5%B7%A2%E8%9B%8B%E7%99%BD%E8%B4%A8%E7%BB%84%E6%9C%80%E6%96%B0%E5%88%86%E6%9E%90%5C20120221_rmx%5Cannotation%5Crmx2_kegg%5Crmx2.htm#gene53) | 6 (1.52%) | ko04114 |
| 54 | [Hypertrophic cardiomyopathy (HCM)](../../../../H:%5CP%E8%9B%8B%E7%99%BD%E8%B4%A8%E7%BB%84%E5%AD%A6%E6%96%87%E7%AB%A0%5C20120515%E5%89%AF%E6%80%A7%E8%85%BA%E5%92%8C%E7%B2%BE%E5%B7%A2%E8%9B%8B%E7%99%BD%E8%B4%A8%E7%BB%84%E6%9C%80%E6%96%B0%E5%88%86%E6%9E%90%5C20120221_rmx%5Cannotation%5Crmx2_kegg%5Crmx2.htm#gene54) | 6 (1.52%) | ko05410 |
| 55 | [Hepatitis C](../../../../H:%5CP%E8%9B%8B%E7%99%BD%E8%B4%A8%E7%BB%84%E5%AD%A6%E6%96%87%E7%AB%A0%5C20120515%E5%89%AF%E6%80%A7%E8%85%BA%E5%92%8C%E7%B2%BE%E5%B7%A2%E8%9B%8B%E7%99%BD%E8%B4%A8%E7%BB%84%E6%9C%80%E6%96%B0%E5%88%86%E6%9E%90%5C20120221_rmx%5Cannotation%5Crmx2_kegg%5Crmx2.htm#gene55) | 5 (1.27%) | ko05160 |
| 56 | [Systemic lupus erythematosus](../../../../H:%5CP%E8%9B%8B%E7%99%BD%E8%B4%A8%E7%BB%84%E5%AD%A6%E6%96%87%E7%AB%A0%5C20120515%E5%89%AF%E6%80%A7%E8%85%BA%E5%92%8C%E7%B2%BE%E5%B7%A2%E8%9B%8B%E7%99%BD%E8%B4%A8%E7%BB%84%E6%9C%80%E6%96%B0%E5%88%86%E6%9E%90%5C20120221_rmx%5Cannotation%5Crmx2_kegg%5Crmx2.htm#gene56) | 5 (1.27%) | ko05322 |
| 57 | [mRNA surveillance pathway](../../../../H:%5CP%E8%9B%8B%E7%99%BD%E8%B4%A8%E7%BB%84%E5%AD%A6%E6%96%87%E7%AB%A0%5C20120515%E5%89%AF%E6%80%A7%E8%85%BA%E5%92%8C%E7%B2%BE%E5%B7%A2%E8%9B%8B%E7%99%BD%E8%B4%A8%E7%BB%84%E6%9C%80%E6%96%B0%E5%88%86%E6%9E%90%5C20120221_rmx%5Cannotation%5Crmx2_kegg%5Crmx2.htm#gene57) | 5 (1.27%) | ko03015 |
| 58 | [Valine, leucine and isoleucine degradation](../../../../H:%5CP%E8%9B%8B%E7%99%BD%E8%B4%A8%E7%BB%84%E5%AD%A6%E6%96%87%E7%AB%A0%5C20120515%E5%89%AF%E6%80%A7%E8%85%BA%E5%92%8C%E7%B2%BE%E5%B7%A2%E8%9B%8B%E7%99%BD%E8%B4%A8%E7%BB%84%E6%9C%80%E6%96%B0%E5%88%86%E6%9E%90%5C20120221_rmx%5Cannotation%5Crmx2_kegg%5Crmx2.htm#gene58) | 5 (1.27%) | ko00280 |
| 59 | [Vibrio cholerae infection](../../../../H:%5CP%E8%9B%8B%E7%99%BD%E8%B4%A8%E7%BB%84%E5%AD%A6%E6%96%87%E7%AB%A0%5C20120515%E5%89%AF%E6%80%A7%E8%85%BA%E5%92%8C%E7%B2%BE%E5%B7%A2%E8%9B%8B%E7%99%BD%E8%B4%A8%E7%BB%84%E6%9C%80%E6%96%B0%E5%88%86%E6%9E%90%5C20120221_rmx%5Cannotation%5Crmx2_kegg%5Crmx2.htm#gene59) | 5 (1.27%) | ko05110 |
| 60 | [Other glycan degradation](../../../../H:%5CP%E8%9B%8B%E7%99%BD%E8%B4%A8%E7%BB%84%E5%AD%A6%E6%96%87%E7%AB%A0%5C20120515%E5%89%AF%E6%80%A7%E8%85%BA%E5%92%8C%E7%B2%BE%E5%B7%A2%E8%9B%8B%E7%99%BD%E8%B4%A8%E7%BB%84%E6%9C%80%E6%96%B0%E5%88%86%E6%9E%90%5C20120221_rmx%5Cannotation%5Crmx2_kegg%5Crmx2.htm#gene60) | 5 (1.27%) | ko00511 |
| 61 | [Ascorbate and aldarate metabolism](../../../../H:%5CP%E8%9B%8B%E7%99%BD%E8%B4%A8%E7%BB%84%E5%AD%A6%E6%96%87%E7%AB%A0%5C20120515%E5%89%AF%E6%80%A7%E8%85%BA%E5%92%8C%E7%B2%BE%E5%B7%A2%E8%9B%8B%E7%99%BD%E8%B4%A8%E7%BB%84%E6%9C%80%E6%96%B0%E5%88%86%E6%9E%90%5C20120221_rmx%5Cannotation%5Crmx2_kegg%5Crmx2.htm#gene61) | 5 (1.27%) | ko00053 |
| 62 | [Arrhythmogenic right ventricular cardiomyopathy (ARVC)](../../../../H:%5CP%E8%9B%8B%E7%99%BD%E8%B4%A8%E7%BB%84%E5%AD%A6%E6%96%87%E7%AB%A0%5C20120515%E5%89%AF%E6%80%A7%E8%85%BA%E5%92%8C%E7%B2%BE%E5%B7%A2%E8%9B%8B%E7%99%BD%E8%B4%A8%E7%BB%84%E6%9C%80%E6%96%B0%E5%88%86%E6%9E%90%5C20120221_rmx%5Cannotation%5Crmx2_kegg%5Crmx2.htm#gene62) | 5 (1.27%) | ko05412 |
| 63 | [Proximal tubule bicarbonate reclamation](../../../../H:%5CP%E8%9B%8B%E7%99%BD%E8%B4%A8%E7%BB%84%E5%AD%A6%E6%96%87%E7%AB%A0%5C20120515%E5%89%AF%E6%80%A7%E8%85%BA%E5%92%8C%E7%B2%BE%E5%B7%A2%E8%9B%8B%E7%99%BD%E8%B4%A8%E7%BB%84%E6%9C%80%E6%96%B0%E5%88%86%E6%9E%90%5C20120221_rmx%5Cannotation%5Crmx2_kegg%5Crmx2.htm#gene63) | 5 (1.27%) | ko04964 |
| 64 | [Drug metabolism - other enzymes](../../../../H:%5CP%E8%9B%8B%E7%99%BD%E8%B4%A8%E7%BB%84%E5%AD%A6%E6%96%87%E7%AB%A0%5C20120515%E5%89%AF%E6%80%A7%E8%85%BA%E5%92%8C%E7%B2%BE%E5%B7%A2%E8%9B%8B%E7%99%BD%E8%B4%A8%E7%BB%84%E6%9C%80%E6%96%B0%E5%88%86%E6%9E%90%5C20120221_rmx%5Cannotation%5Crmx2_kegg%5Crmx2.htm#gene64) | 4 (1.02%) | ko00983 |
| 65 | [Selenocompound metabolism](../../../../H:%5CP%E8%9B%8B%E7%99%BD%E8%B4%A8%E7%BB%84%E5%AD%A6%E6%96%87%E7%AB%A0%5C20120515%E5%89%AF%E6%80%A7%E8%85%BA%E5%92%8C%E7%B2%BE%E5%B7%A2%E8%9B%8B%E7%99%BD%E8%B4%A8%E7%BB%84%E6%9C%80%E6%96%B0%E5%88%86%E6%9E%90%5C20120221_rmx%5Cannotation%5Crmx2_kegg%5Crmx2.htm#gene65) | 4 (1.02%) | ko00450 |
| 66 | [Vascular smooth muscle contraction](../../../../H:%5CP%E8%9B%8B%E7%99%BD%E8%B4%A8%E7%BB%84%E5%AD%A6%E6%96%87%E7%AB%A0%5C20120515%E5%89%AF%E6%80%A7%E8%85%BA%E5%92%8C%E7%B2%BE%E5%B7%A2%E8%9B%8B%E7%99%BD%E8%B4%A8%E7%BB%84%E6%9C%80%E6%96%B0%E5%88%86%E6%9E%90%5C20120221_rmx%5Cannotation%5Crmx2_kegg%5Crmx2.htm#gene66) | 4 (1.02%) | ko04270 |
| 67 | [Histidine metabolism](../../../../H:%5CP%E8%9B%8B%E7%99%BD%E8%B4%A8%E7%BB%84%E5%AD%A6%E6%96%87%E7%AB%A0%5C20120515%E5%89%AF%E6%80%A7%E8%85%BA%E5%92%8C%E7%B2%BE%E5%B7%A2%E8%9B%8B%E7%99%BD%E8%B4%A8%E7%BB%84%E6%9C%80%E6%96%B0%E5%88%86%E6%9E%90%5C20120221_rmx%5Cannotation%5Crmx2_kegg%5Crmx2.htm#gene67) | 4 (1.02%) | ko00340 |
| 68 | [Metabolism of xenobiotics by cytochrome P450](../../../../H:%5CP%E8%9B%8B%E7%99%BD%E8%B4%A8%E7%BB%84%E5%AD%A6%E6%96%87%E7%AB%A0%5C20120515%E5%89%AF%E6%80%A7%E8%85%BA%E5%92%8C%E7%B2%BE%E5%B7%A2%E8%9B%8B%E7%99%BD%E8%B4%A8%E7%BB%84%E6%9C%80%E6%96%B0%E5%88%86%E6%9E%90%5C20120221_rmx%5Cannotation%5Crmx2_kegg%5Crmx2.htm#gene68) | 4 (1.02%) | ko00980 |
| 69 | [Ribosome biogenesis in eukaryotes](../../../../H:%5CP%E8%9B%8B%E7%99%BD%E8%B4%A8%E7%BB%84%E5%AD%A6%E6%96%87%E7%AB%A0%5C20120515%E5%89%AF%E6%80%A7%E8%85%BA%E5%92%8C%E7%B2%BE%E5%B7%A2%E8%9B%8B%E7%99%BD%E8%B4%A8%E7%BB%84%E6%9C%80%E6%96%B0%E5%88%86%E6%9E%90%5C20120221_rmx%5Cannotation%5Crmx2_kegg%5Crmx2.htm#gene69) | 4 (1.02%) | ko03008 |
| 70 | [Sphingolipid metabolism](../../../../H:%5CP%E8%9B%8B%E7%99%BD%E8%B4%A8%E7%BB%84%E5%AD%A6%E6%96%87%E7%AB%A0%5C20120515%E5%89%AF%E6%80%A7%E8%85%BA%E5%92%8C%E7%B2%BE%E5%B7%A2%E8%9B%8B%E7%99%BD%E8%B4%A8%E7%BB%84%E6%9C%80%E6%96%B0%E5%88%86%E6%9E%90%5C20120221_rmx%5Cannotation%5Crmx2_kegg%5Crmx2.htm#gene70) | 4 (1.02%) | ko00600 |
| 71 | [beta-Alanine metabolism](../../../../H:%5CP%E8%9B%8B%E7%99%BD%E8%B4%A8%E7%BB%84%E5%AD%A6%E6%96%87%E7%AB%A0%5C20120515%E5%89%AF%E6%80%A7%E8%85%BA%E5%92%8C%E7%B2%BE%E5%B7%A2%E8%9B%8B%E7%99%BD%E8%B4%A8%E7%BB%84%E6%9C%80%E6%96%B0%E5%88%86%E6%9E%90%5C20120221_rmx%5Cannotation%5Crmx2_kegg%5Crmx2.htm#gene71) | 4 (1.02%) | ko00410 |
| 72 | [Drug metabolism - cytochrome P450](../../../../H:%5CP%E8%9B%8B%E7%99%BD%E8%B4%A8%E7%BB%84%E5%AD%A6%E6%96%87%E7%AB%A0%5C20120515%E5%89%AF%E6%80%A7%E8%85%BA%E5%92%8C%E7%B2%BE%E5%B7%A2%E8%9B%8B%E7%99%BD%E8%B4%A8%E7%BB%84%E6%9C%80%E6%96%B0%E5%88%86%E6%9E%90%5C20120221_rmx%5Cannotation%5Crmx2_kegg%5Crmx2.htm#gene72) | 4 (1.02%) | ko00982 |
| 73 | [Glycine, serine and threonine metabolism](../../../../H:%5CP%E8%9B%8B%E7%99%BD%E8%B4%A8%E7%BB%84%E5%AD%A6%E6%96%87%E7%AB%A0%5C20120515%E5%89%AF%E6%80%A7%E8%85%BA%E5%92%8C%E7%B2%BE%E5%B7%A2%E8%9B%8B%E7%99%BD%E8%B4%A8%E7%BB%84%E6%9C%80%E6%96%B0%E5%88%86%E6%9E%90%5C20120221_rmx%5Cannotation%5Crmx2_kegg%5Crmx2.htm#gene73) | 4 (1.02%) | ko00260 |
| 74 | [Insulin signaling pathway](../../../../H:%5CP%E8%9B%8B%E7%99%BD%E8%B4%A8%E7%BB%84%E5%AD%A6%E6%96%87%E7%AB%A0%5C20120515%E5%89%AF%E6%80%A7%E8%85%BA%E5%92%8C%E7%B2%BE%E5%B7%A2%E8%9B%8B%E7%99%BD%E8%B4%A8%E7%BB%84%E6%9C%80%E6%96%B0%E5%88%86%E6%9E%90%5C20120221_rmx%5Cannotation%5Crmx2_kegg%5Crmx2.htm#gene74) | 4 (1.02%) | ko04910 |
| 75 | [Lysine degradation](../../../../H:%5CP%E8%9B%8B%E7%99%BD%E8%B4%A8%E7%BB%84%E5%AD%A6%E6%96%87%E7%AB%A0%5C20120515%E5%89%AF%E6%80%A7%E8%85%BA%E5%92%8C%E7%B2%BE%E5%B7%A2%E8%9B%8B%E7%99%BD%E8%B4%A8%E7%BB%84%E6%9C%80%E6%96%B0%E5%88%86%E6%9E%90%5C20120221_rmx%5Cannotation%5Crmx2_kegg%5Crmx2.htm#gene75) | 4 (1.02%) | ko00310 |
| 76 | [Huntington's disease](../../../../H:%5CP%E8%9B%8B%E7%99%BD%E8%B4%A8%E7%BB%84%E5%AD%A6%E6%96%87%E7%AB%A0%5C20120515%E5%89%AF%E6%80%A7%E8%85%BA%E5%92%8C%E7%B2%BE%E5%B7%A2%E8%9B%8B%E7%99%BD%E8%B4%A8%E7%BB%84%E6%9C%80%E6%96%B0%E5%88%86%E6%9E%90%5C20120221_rmx%5Cannotation%5Crmx2_kegg%5Crmx2.htm#gene76) | 4 (1.02%) | ko05016 |
| 77 | [Fatty acid metabolism](../../../../H:%5CP%E8%9B%8B%E7%99%BD%E8%B4%A8%E7%BB%84%E5%AD%A6%E6%96%87%E7%AB%A0%5C20120515%E5%89%AF%E6%80%A7%E8%85%BA%E5%92%8C%E7%B2%BE%E5%B7%A2%E8%9B%8B%E7%99%BD%E8%B4%A8%E7%BB%84%E6%9C%80%E6%96%B0%E5%88%86%E6%9E%90%5C20120221_rmx%5Cannotation%5Crmx2_kegg%5Crmx2.htm#gene77) | 4 (1.02%) | ko00071 |
| 78 | [Nitrogen metabolism](../../../../H:%5CP%E8%9B%8B%E7%99%BD%E8%B4%A8%E7%BB%84%E5%AD%A6%E6%96%87%E7%AB%A0%5C20120515%E5%89%AF%E6%80%A7%E8%85%BA%E5%92%8C%E7%B2%BE%E5%B7%A2%E8%9B%8B%E7%99%BD%E8%B4%A8%E7%BB%84%E6%9C%80%E6%96%B0%E5%88%86%E6%9E%90%5C20120221_rmx%5Cannotation%5Crmx2_kegg%5Crmx2.htm#gene78) | 4 (1.02%) | ko00910 |
| 79 | [Vasopressin-regulated water reabsorption](../../../../H:%5CP%E8%9B%8B%E7%99%BD%E8%B4%A8%E7%BB%84%E5%AD%A6%E6%96%87%E7%AB%A0%5C20120515%E5%89%AF%E6%80%A7%E8%85%BA%E5%92%8C%E7%B2%BE%E5%B7%A2%E8%9B%8B%E7%99%BD%E8%B4%A8%E7%BB%84%E6%9C%80%E6%96%B0%E5%88%86%E6%9E%90%5C20120221_rmx%5Cannotation%5Crmx2_kegg%5Crmx2.htm#gene79) | 4 (1.02%) | ko04962 |
| 80 | [Prion diseases](../../../../H:%5CP%E8%9B%8B%E7%99%BD%E8%B4%A8%E7%BB%84%E5%AD%A6%E6%96%87%E7%AB%A0%5C20120515%E5%89%AF%E6%80%A7%E8%85%BA%E5%92%8C%E7%B2%BE%E5%B7%A2%E8%9B%8B%E7%99%BD%E8%B4%A8%E7%BB%84%E6%9C%80%E6%96%B0%E5%88%86%E6%9E%90%5C20120221_rmx%5Cannotation%5Crmx2_kegg%5Crmx2.htm#gene80) | 4 (1.02%) | ko05020 |
| 81 | [Glycerolipid metabolism](../../../../H:%5CP%E8%9B%8B%E7%99%BD%E8%B4%A8%E7%BB%84%E5%AD%A6%E6%96%87%E7%AB%A0%5C20120515%E5%89%AF%E6%80%A7%E8%85%BA%E5%92%8C%E7%B2%BE%E5%B7%A2%E8%9B%8B%E7%99%BD%E8%B4%A8%E7%BB%84%E6%9C%80%E6%96%B0%E5%88%86%E6%9E%90%5C20120221_rmx%5Cannotation%5Crmx2_kegg%5Crmx2.htm#gene81) | 4 (1.02%) | ko00561 |
| 82 | [Amyotrophic lateral sclerosis (ALS)](../../../../H:%5CP%E8%9B%8B%E7%99%BD%E8%B4%A8%E7%BB%84%E5%AD%A6%E6%96%87%E7%AB%A0%5C20120515%E5%89%AF%E6%80%A7%E8%85%BA%E5%92%8C%E7%B2%BE%E5%B7%A2%E8%9B%8B%E7%99%BD%E8%B4%A8%E7%BB%84%E6%9C%80%E6%96%B0%E5%88%86%E6%9E%90%5C20120221_rmx%5Cannotation%5Crmx2_kegg%5Crmx2.htm#gene82) | 4 (1.02%) | ko05014 |
| 83 | [Phototransduction - fly](../../../../H:%5CP%E8%9B%8B%E7%99%BD%E8%B4%A8%E7%BB%84%E5%AD%A6%E6%96%87%E7%AB%A0%5C20120515%E5%89%AF%E6%80%A7%E8%85%BA%E5%92%8C%E7%B2%BE%E5%B7%A2%E8%9B%8B%E7%99%BD%E8%B4%A8%E7%BB%84%E6%9C%80%E6%96%B0%E5%88%86%E6%9E%90%5C20120221_rmx%5Cannotation%5Crmx2_kegg%5Crmx2.htm#gene83) | 4 (1.02%) | ko04745 |
| 84 | [Cell cycle](../../../../H:%5CP%E8%9B%8B%E7%99%BD%E8%B4%A8%E7%BB%84%E5%AD%A6%E6%96%87%E7%AB%A0%5C20120515%E5%89%AF%E6%80%A7%E8%85%BA%E5%92%8C%E7%B2%BE%E5%B7%A2%E8%9B%8B%E7%99%BD%E8%B4%A8%E7%BB%84%E6%9C%80%E6%96%B0%E5%88%86%E6%9E%90%5C20120221_rmx%5Cannotation%5Crmx2_kegg%5Crmx2.htm#gene84) | 4 (1.02%) | ko04110 |
| 85 | [PPAR signaling pathway](../../../../H:%5CP%E8%9B%8B%E7%99%BD%E8%B4%A8%E7%BB%84%E5%AD%A6%E6%96%87%E7%AB%A0%5C20120515%E5%89%AF%E6%80%A7%E8%85%BA%E5%92%8C%E7%B2%BE%E5%B7%A2%E8%9B%8B%E7%99%BD%E8%B4%A8%E7%BB%84%E6%9C%80%E6%96%B0%E5%88%86%E6%9E%90%5C20120221_rmx%5Cannotation%5Crmx2_kegg%5Crmx2.htm#gene85) | 3 (0.76%) | ko03320 |
| 86 | [Limonene and pinene degradation](../../../../H:%5CP%E8%9B%8B%E7%99%BD%E8%B4%A8%E7%BB%84%E5%AD%A6%E6%96%87%E7%AB%A0%5C20120515%E5%89%AF%E6%80%A7%E8%85%BA%E5%92%8C%E7%B2%BE%E5%B7%A2%E8%9B%8B%E7%99%BD%E8%B4%A8%E7%BB%84%E6%9C%80%E6%96%B0%E5%88%86%E6%9E%90%5C20120221_rmx%5Cannotation%5Crmx2_kegg%5Crmx2.htm#gene86) | 3 (0.76%) | ko00903 |
| 87 | [NOD-like receptor signaling pathway](../../../../H:%5CP%E8%9B%8B%E7%99%BD%E8%B4%A8%E7%BB%84%E5%AD%A6%E6%96%87%E7%AB%A0%5C20120515%E5%89%AF%E6%80%A7%E8%85%BA%E5%92%8C%E7%B2%BE%E5%B7%A2%E8%9B%8B%E7%99%BD%E8%B4%A8%E7%BB%84%E6%9C%80%E6%96%B0%E5%88%86%E6%9E%90%5C20120221_rmx%5Cannotation%5Crmx2_kegg%5Crmx2.htm#gene87) | 3 (0.76%) | ko04621 |
| 88 | [Neuroactive ligand-receptor interaction](../../../../H:%5CP%E8%9B%8B%E7%99%BD%E8%B4%A8%E7%BB%84%E5%AD%A6%E6%96%87%E7%AB%A0%5C20120515%E5%89%AF%E6%80%A7%E8%85%BA%E5%92%8C%E7%B2%BE%E5%B7%A2%E8%9B%8B%E7%99%BD%E8%B4%A8%E7%BB%84%E6%9C%80%E6%96%B0%E5%88%86%E6%9E%90%5C20120221_rmx%5Cannotation%5Crmx2_kegg%5Crmx2.htm#gene88) | 3 (0.76%) | ko04080 |
| 89 | [Glycerophospholipid metabolism](../../../../H:%5CP%E8%9B%8B%E7%99%BD%E8%B4%A8%E7%BB%84%E5%AD%A6%E6%96%87%E7%AB%A0%5C20120515%E5%89%AF%E6%80%A7%E8%85%BA%E5%92%8C%E7%B2%BE%E5%B7%A2%E8%9B%8B%E7%99%BD%E8%B4%A8%E7%BB%84%E6%9C%80%E6%96%B0%E5%88%86%E6%9E%90%5C20120221_rmx%5Cannotation%5Crmx2_kegg%5Crmx2.htm#gene89) | 3 (0.76%) | ko00564 |
| 90 | [Glycosphingolipid biosynthesis - globo series](../../../../H:%5CP%E8%9B%8B%E7%99%BD%E8%B4%A8%E7%BB%84%E5%AD%A6%E6%96%87%E7%AB%A0%5C20120515%E5%89%AF%E6%80%A7%E8%85%BA%E5%92%8C%E7%B2%BE%E5%B7%A2%E8%9B%8B%E7%99%BD%E8%B4%A8%E7%BB%84%E6%9C%80%E6%96%B0%E5%88%86%E6%9E%90%5C20120221_rmx%5Cannotation%5Crmx2_kegg%5Crmx2.htm#gene90) | 3 (0.76%) | ko00603 |
| 91 | [Collecting duct acid secretion](../../../../H:%5CP%E8%9B%8B%E7%99%BD%E8%B4%A8%E7%BB%84%E5%AD%A6%E6%96%87%E7%AB%A0%5C20120515%E5%89%AF%E6%80%A7%E8%85%BA%E5%92%8C%E7%B2%BE%E5%B7%A2%E8%9B%8B%E7%99%BD%E8%B4%A8%E7%BB%84%E6%9C%80%E6%96%B0%E5%88%86%E6%9E%90%5C20120221_rmx%5Cannotation%5Crmx2_kegg%5Crmx2.htm#gene91) | 3 (0.76%) | ko04966 |
| 92 | [MAPK signaling pathway](../../../../H:%5CP%E8%9B%8B%E7%99%BD%E8%B4%A8%E7%BB%84%E5%AD%A6%E6%96%87%E7%AB%A0%5C20120515%E5%89%AF%E6%80%A7%E8%85%BA%E5%92%8C%E7%B2%BE%E5%B7%A2%E8%9B%8B%E7%99%BD%E8%B4%A8%E7%BB%84%E6%9C%80%E6%96%B0%E5%88%86%E6%9E%90%5C20120221_rmx%5Cannotation%5Crmx2_kegg%5Crmx2.htm#gene92) | 3 (0.76%) | ko04010 |
| 93 | [Glycosaminoglycan biosynthesis - chondroitin sulfate](../../../../H:%5CP%E8%9B%8B%E7%99%BD%E8%B4%A8%E7%BB%84%E5%AD%A6%E6%96%87%E7%AB%A0%5C20120515%E5%89%AF%E6%80%A7%E8%85%BA%E5%92%8C%E7%B2%BE%E5%B7%A2%E8%9B%8B%E7%99%BD%E8%B4%A8%E7%BB%84%E6%9C%80%E6%96%B0%E5%88%86%E6%9E%90%5C20120221_rmx%5Cannotation%5Crmx2_kegg%5Crmx2.htm#gene93) | 3 (0.76%) | ko00532 |
| 94 | [Phenylalanine metabolism](../../../../H:%5CP%E8%9B%8B%E7%99%BD%E8%B4%A8%E7%BB%84%E5%AD%A6%E6%96%87%E7%AB%A0%5C20120515%E5%89%AF%E6%80%A7%E8%85%BA%E5%92%8C%E7%B2%BE%E5%B7%A2%E8%9B%8B%E7%99%BD%E8%B4%A8%E7%BB%84%E6%9C%80%E6%96%B0%E5%88%86%E6%9E%90%5C20120221_rmx%5Cannotation%5Crmx2_kegg%5Crmx2.htm#gene94) | 3 (0.76%) | ko00360 |
| 95 | [Pyrimidine metabolism](../../../../H:%5CP%E8%9B%8B%E7%99%BD%E8%B4%A8%E7%BB%84%E5%AD%A6%E6%96%87%E7%AB%A0%5C20120515%E5%89%AF%E6%80%A7%E8%85%BA%E5%92%8C%E7%B2%BE%E5%B7%A2%E8%9B%8B%E7%99%BD%E8%B4%A8%E7%BB%84%E6%9C%80%E6%96%B0%E5%88%86%E6%9E%90%5C20120221_rmx%5Cannotation%5Crmx2_kegg%5Crmx2.htm#gene95) | 3 (0.76%) | ko00240 |
| 96 | [Renin-angiotensin system](../../../../H:%5CP%E8%9B%8B%E7%99%BD%E8%B4%A8%E7%BB%84%E5%AD%A6%E6%96%87%E7%AB%A0%5C20120515%E5%89%AF%E6%80%A7%E8%85%BA%E5%92%8C%E7%B2%BE%E5%B7%A2%E8%9B%8B%E7%99%BD%E8%B4%A8%E7%BB%84%E6%9C%80%E6%96%B0%E5%88%86%E6%9E%90%5C20120221_rmx%5Cannotation%5Crmx2_kegg%5Crmx2.htm#gene96) | 3 (0.76%) | ko04614 |
| 97 | [RNA degradation](../../../../H:%5CP%E8%9B%8B%E7%99%BD%E8%B4%A8%E7%BB%84%E5%AD%A6%E6%96%87%E7%AB%A0%5C20120515%E5%89%AF%E6%80%A7%E8%85%BA%E5%92%8C%E7%B2%BE%E5%B7%A2%E8%9B%8B%E7%99%BD%E8%B4%A8%E7%BB%84%E6%9C%80%E6%96%B0%E5%88%86%E6%9E%90%5C20120221_rmx%5Cannotation%5Crmx2_kegg%5Crmx2.htm#gene97) | 3 (0.76%) | ko03018 |
| 98 | [Renal cell carcinoma](../../../../H:%5CP%E8%9B%8B%E7%99%BD%E8%B4%A8%E7%BB%84%E5%AD%A6%E6%96%87%E7%AB%A0%5C20120515%E5%89%AF%E6%80%A7%E8%85%BA%E5%92%8C%E7%B2%BE%E5%B7%A2%E8%9B%8B%E7%99%BD%E8%B4%A8%E7%BB%84%E6%9C%80%E6%96%B0%E5%88%86%E6%9E%90%5C20120221_rmx%5Cannotation%5Crmx2_kegg%5Crmx2.htm#gene98) | 2 (0.51%) | ko05211 |
| 99 | [Glycosphingolipid biosynthesis - ganglio series](../../../../H:%5CP%E8%9B%8B%E7%99%BD%E8%B4%A8%E7%BB%84%E5%AD%A6%E6%96%87%E7%AB%A0%5C20120515%E5%89%AF%E6%80%A7%E8%85%BA%E5%92%8C%E7%B2%BE%E5%B7%A2%E8%9B%8B%E7%99%BD%E8%B4%A8%E7%BB%84%E6%9C%80%E6%96%B0%E5%88%86%E6%9E%90%5C20120221_rmx%5Cannotation%5Crmx2_kegg%5Crmx2.htm#gene99) | 2 (0.51%) | ko00604 |
| 100 | [Bile secretion](../../../../H:%5CP%E8%9B%8B%E7%99%BD%E8%B4%A8%E7%BB%84%E5%AD%A6%E6%96%87%E7%AB%A0%5C20120515%E5%89%AF%E6%80%A7%E8%85%BA%E5%92%8C%E7%B2%BE%E5%B7%A2%E8%9B%8B%E7%99%BD%E8%B4%A8%E7%BB%84%E6%9C%80%E6%96%B0%E5%88%86%E6%9E%90%5C20120221_rmx%5Cannotation%5Crmx2_kegg%5Crmx2.htm#gene100) | 2 (0.51%) | ko04976 |
| 101 | [Axon guidance](../../../../H:%5CP%E8%9B%8B%E7%99%BD%E8%B4%A8%E7%BB%84%E5%AD%A6%E6%96%87%E7%AB%A0%5C20120515%E5%89%AF%E6%80%A7%E8%85%BA%E5%92%8C%E7%B2%BE%E5%B7%A2%E8%9B%8B%E7%99%BD%E8%B4%A8%E7%BB%84%E6%9C%80%E6%96%B0%E5%88%86%E6%9E%90%5C20120221_rmx%5Cannotation%5Crmx2_kegg%5Crmx2.htm#gene101) | 2 (0.51%) | ko04360 |
| 102 | [Protein export](../../../../H:%5CP%E8%9B%8B%E7%99%BD%E8%B4%A8%E7%BB%84%E5%AD%A6%E6%96%87%E7%AB%A0%5C20120515%E5%89%AF%E6%80%A7%E8%85%BA%E5%92%8C%E7%B2%BE%E5%B7%A2%E8%9B%8B%E7%99%BD%E8%B4%A8%E7%BB%84%E6%9C%80%E6%96%B0%E5%88%86%E6%9E%90%5C20120221_rmx%5Cannotation%5Crmx2_kegg%5Crmx2.htm#gene102) | 2 (0.51%) | ko03060 |
| 103 | [Glycosaminoglycan degradation](../../../../H:%5CP%E8%9B%8B%E7%99%BD%E8%B4%A8%E7%BB%84%E5%AD%A6%E6%96%87%E7%AB%A0%5C20120515%E5%89%AF%E6%80%A7%E8%85%BA%E5%92%8C%E7%B2%BE%E5%B7%A2%E8%9B%8B%E7%99%BD%E8%B4%A8%E7%BB%84%E6%9C%80%E6%96%B0%E5%88%86%E6%9E%90%5C20120221_rmx%5Cannotation%5Crmx2_kegg%5Crmx2.htm#gene103) | 2 (0.51%) | ko00531 |
| 104 | [Chemokine signaling pathway](../../../../H:%5CP%E8%9B%8B%E7%99%BD%E8%B4%A8%E7%BB%84%E5%AD%A6%E6%96%87%E7%AB%A0%5C20120515%E5%89%AF%E6%80%A7%E8%85%BA%E5%92%8C%E7%B2%BE%E5%B7%A2%E8%9B%8B%E7%99%BD%E8%B4%A8%E7%BB%84%E6%9C%80%E6%96%B0%E5%88%86%E6%9E%90%5C20120221_rmx%5Cannotation%5Crmx2_kegg%5Crmx2.htm#gene104) | 2 (0.51%) | ko04062 |
| 105 | [Toxoplasmosis](../../../../H:%5CP%E8%9B%8B%E7%99%BD%E8%B4%A8%E7%BB%84%E5%AD%A6%E6%96%87%E7%AB%A0%5C20120515%E5%89%AF%E6%80%A7%E8%85%BA%E5%92%8C%E7%B2%BE%E5%B7%A2%E8%9B%8B%E7%99%BD%E8%B4%A8%E7%BB%84%E6%9C%80%E6%96%B0%E5%88%86%E6%9E%90%5C20120221_rmx%5Cannotation%5Crmx2_kegg%5Crmx2.htm#gene105) | 2 (0.51%) | ko05145 |
| 106 | [Cardiac muscle contraction](../../../../H:%5CP%E8%9B%8B%E7%99%BD%E8%B4%A8%E7%BB%84%E5%AD%A6%E6%96%87%E7%AB%A0%5C20120515%E5%89%AF%E6%80%A7%E8%85%BA%E5%92%8C%E7%B2%BE%E5%B7%A2%E8%9B%8B%E7%99%BD%E8%B4%A8%E7%BB%84%E6%9C%80%E6%96%B0%E5%88%86%E6%9E%90%5C20120221_rmx%5Cannotation%5Crmx2_kegg%5Crmx2.htm#gene106) | 2 (0.51%) | ko04260 |
| 107 | [Sulfur metabolism](../../../../H:%5CP%E8%9B%8B%E7%99%BD%E8%B4%A8%E7%BB%84%E5%AD%A6%E6%96%87%E7%AB%A0%5C20120515%E5%89%AF%E6%80%A7%E8%85%BA%E5%92%8C%E7%B2%BE%E5%B7%A2%E8%9B%8B%E7%99%BD%E8%B4%A8%E7%BB%84%E6%9C%80%E6%96%B0%E5%88%86%E6%9E%90%5C20120221_rmx%5Cannotation%5Crmx2_kegg%5Crmx2.htm#gene107) | 2 (0.51%) | ko00920 |
| 108 | [Fc gamma R-mediated phagocytosis](../../../../H:%5CP%E8%9B%8B%E7%99%BD%E8%B4%A8%E7%BB%84%E5%AD%A6%E6%96%87%E7%AB%A0%5C20120515%E5%89%AF%E6%80%A7%E8%85%BA%E5%92%8C%E7%B2%BE%E5%B7%A2%E8%9B%8B%E7%99%BD%E8%B4%A8%E7%BB%84%E6%9C%80%E6%96%B0%E5%88%86%E6%9E%90%5C20120221_rmx%5Cannotation%5Crmx2_kegg%5Crmx2.htm#gene108) | 2 (0.51%) | ko04666 |
| 109 | [Parkinson's disease](../../../../H:%5CP%E8%9B%8B%E7%99%BD%E8%B4%A8%E7%BB%84%E5%AD%A6%E6%96%87%E7%AB%A0%5C20120515%E5%89%AF%E6%80%A7%E8%85%BA%E5%92%8C%E7%B2%BE%E5%B7%A2%E8%9B%8B%E7%99%BD%E8%B4%A8%E7%BB%84%E6%9C%80%E6%96%B0%E5%88%86%E6%9E%90%5C20120221_rmx%5Cannotation%5Crmx2_kegg%5Crmx2.htm#gene109) | 2 (0.51%) | ko05012 |
| 110 | [Oxidative phosphorylation](../../../../H:%5CP%E8%9B%8B%E7%99%BD%E8%B4%A8%E7%BB%84%E5%AD%A6%E6%96%87%E7%AB%A0%5C20120515%E5%89%AF%E6%80%A7%E8%85%BA%E5%92%8C%E7%B2%BE%E5%B7%A2%E8%9B%8B%E7%99%BD%E8%B4%A8%E7%BB%84%E6%9C%80%E6%96%B0%E5%88%86%E6%9E%90%5C20120221_rmx%5Cannotation%5Crmx2_kegg%5Crmx2.htm#gene110) | 2 (0.51%) | ko00190 |
| 111 | [Inositol phosphate metabolism](../../../../H:%5CP%E8%9B%8B%E7%99%BD%E8%B4%A8%E7%BB%84%E5%AD%A6%E6%96%87%E7%AB%A0%5C20120515%E5%89%AF%E6%80%A7%E8%85%BA%E5%92%8C%E7%B2%BE%E5%B7%A2%E8%9B%8B%E7%99%BD%E8%B4%A8%E7%BB%84%E6%9C%80%E6%96%B0%E5%88%86%E6%9E%90%5C20120221_rmx%5Cannotation%5Crmx2_kegg%5Crmx2.htm#gene111) | 2 (0.51%) | ko00562 |
| 112 | [Hematopoietic cell lineage](../../../../H:%5CP%E8%9B%8B%E7%99%BD%E8%B4%A8%E7%BB%84%E5%AD%A6%E6%96%87%E7%AB%A0%5C20120515%E5%89%AF%E6%80%A7%E8%85%BA%E5%92%8C%E7%B2%BE%E5%B7%A2%E8%9B%8B%E7%99%BD%E8%B4%A8%E7%BB%84%E6%9C%80%E6%96%B0%E5%88%86%E6%9E%90%5C20120221_rmx%5Cannotation%5Crmx2_kegg%5Crmx2.htm#gene112) | 2 (0.51%) | ko04640 |
| 113 | [Arachidonic acid metabolism](../../../../H:%5CP%E8%9B%8B%E7%99%BD%E8%B4%A8%E7%BB%84%E5%AD%A6%E6%96%87%E7%AB%A0%5C20120515%E5%89%AF%E6%80%A7%E8%85%BA%E5%92%8C%E7%B2%BE%E5%B7%A2%E8%9B%8B%E7%99%BD%E8%B4%A8%E7%BB%84%E6%9C%80%E6%96%B0%E5%88%86%E6%9E%90%5C20120221_rmx%5Cannotation%5Crmx2_kegg%5Crmx2.htm#gene113) | 2 (0.51%) | ko00590 |
| 114 | [Wnt signaling pathway](../../../../H:%5CP%E8%9B%8B%E7%99%BD%E8%B4%A8%E7%BB%84%E5%AD%A6%E6%96%87%E7%AB%A0%5C20120515%E5%89%AF%E6%80%A7%E8%85%BA%E5%92%8C%E7%B2%BE%E5%B7%A2%E8%9B%8B%E7%99%BD%E8%B4%A8%E7%BB%84%E6%9C%80%E6%96%B0%E5%88%86%E6%9E%90%5C20120221_rmx%5Cannotation%5Crmx2_kegg%5Crmx2.htm#gene114) | 2 (0.51%) | ko04310 |
| 115 | [Folate biosynthesis](../../../../H:%5CP%E8%9B%8B%E7%99%BD%E8%B4%A8%E7%BB%84%E5%AD%A6%E6%96%87%E7%AB%A0%5C20120515%E5%89%AF%E6%80%A7%E8%85%BA%E5%92%8C%E7%B2%BE%E5%B7%A2%E8%9B%8B%E7%99%BD%E8%B4%A8%E7%BB%84%E6%9C%80%E6%96%B0%E5%88%86%E6%9E%90%5C20120221_rmx%5Cannotation%5Crmx2_kegg%5Crmx2.htm#gene115) | 2 (0.51%) | ko00790 |
| 116 | [Adipocytokine signaling pathway](../../../../H:%5CP%E8%9B%8B%E7%99%BD%E8%B4%A8%E7%BB%84%E5%AD%A6%E6%96%87%E7%AB%A0%5C20120515%E5%89%AF%E6%80%A7%E8%85%BA%E5%92%8C%E7%B2%BE%E5%B7%A2%E8%9B%8B%E7%99%BD%E8%B4%A8%E7%BB%84%E6%9C%80%E6%96%B0%E5%88%86%E6%9E%90%5C20120221_rmx%5Cannotation%5Crmx2_kegg%5Crmx2.htm#gene116) | 2 (0.51%) | ko04920 |
| 117 | [Alzheimer's disease](../../../../H:%5CP%E8%9B%8B%E7%99%BD%E8%B4%A8%E7%BB%84%E5%AD%A6%E6%96%87%E7%AB%A0%5C20120515%E5%89%AF%E6%80%A7%E8%85%BA%E5%92%8C%E7%B2%BE%E5%B7%A2%E8%9B%8B%E7%99%BD%E8%B4%A8%E7%BB%84%E6%9C%80%E6%96%B0%E5%88%86%E6%9E%90%5C20120221_rmx%5Cannotation%5Crmx2_kegg%5Crmx2.htm#gene117) | 2 (0.51%) | ko05010 |
| 118 | [Phenylalanine, tyrosine and tryptophan biosynthesis](../../../../H:%5CP%E8%9B%8B%E7%99%BD%E8%B4%A8%E7%BB%84%E5%AD%A6%E6%96%87%E7%AB%A0%5C20120515%E5%89%AF%E6%80%A7%E8%85%BA%E5%92%8C%E7%B2%BE%E5%B7%A2%E8%9B%8B%E7%99%BD%E8%B4%A8%E7%BB%84%E6%9C%80%E6%96%B0%E5%88%86%E6%9E%90%5C20120221_rmx%5Cannotation%5Crmx2_kegg%5Crmx2.htm#gene118) | 2 (0.51%) | ko00400 |
| 119 | [Fat digestion and absorption](../../../../H:%5CP%E8%9B%8B%E7%99%BD%E8%B4%A8%E7%BB%84%E5%AD%A6%E6%96%87%E7%AB%A0%5C20120515%E5%89%AF%E6%80%A7%E8%85%BA%E5%92%8C%E7%B2%BE%E5%B7%A2%E8%9B%8B%E7%99%BD%E8%B4%A8%E7%BB%84%E6%9C%80%E6%96%B0%E5%88%86%E6%9E%90%5C20120221_rmx%5Cannotation%5Crmx2_kegg%5Crmx2.htm#gene119) | 2 (0.51%) | ko04975 |
| 120 | [Type II diabetes mellitus](../../../../H:%5CP%E8%9B%8B%E7%99%BD%E8%B4%A8%E7%BB%84%E5%AD%A6%E6%96%87%E7%AB%A0%5C20120515%E5%89%AF%E6%80%A7%E8%85%BA%E5%92%8C%E7%B2%BE%E5%B7%A2%E8%9B%8B%E7%99%BD%E8%B4%A8%E7%BB%84%E6%9C%80%E6%96%B0%E5%88%86%E6%9E%90%5C20120221_rmx%5Cannotation%5Crmx2_kegg%5Crmx2.htm#gene120) | 2 (0.51%) | ko04930 |
| 121 | [Rheumatoid arthritis](../../../../H:%5CP%E8%9B%8B%E7%99%BD%E8%B4%A8%E7%BB%84%E5%AD%A6%E6%96%87%E7%AB%A0%5C20120515%E5%89%AF%E6%80%A7%E8%85%BA%E5%92%8C%E7%B2%BE%E5%B7%A2%E8%9B%8B%E7%99%BD%E8%B4%A8%E7%BB%84%E6%9C%80%E6%96%B0%E5%88%86%E6%9E%90%5C20120221_rmx%5Cannotation%5Crmx2_kegg%5Crmx2.htm#gene121) | 2 (0.51%) | ko05323 |
| 122 | [Long-term potentiation](../../../../H:%5CP%E8%9B%8B%E7%99%BD%E8%B4%A8%E7%BB%84%E5%AD%A6%E6%96%87%E7%AB%A0%5C20120515%E5%89%AF%E6%80%A7%E8%85%BA%E5%92%8C%E7%B2%BE%E5%B7%A2%E8%9B%8B%E7%99%BD%E8%B4%A8%E7%BB%84%E6%9C%80%E6%96%B0%E5%88%86%E6%9E%90%5C20120221_rmx%5Cannotation%5Crmx2_kegg%5Crmx2.htm#gene122) | 2 (0.51%) | ko04720 |
| 123 | [TGF-beta signaling pathway](../../../../H:%5CP%E8%9B%8B%E7%99%BD%E8%B4%A8%E7%BB%84%E5%AD%A6%E6%96%87%E7%AB%A0%5C20120515%E5%89%AF%E6%80%A7%E8%85%BA%E5%92%8C%E7%B2%BE%E5%B7%A2%E8%9B%8B%E7%99%BD%E8%B4%A8%E7%BB%84%E6%9C%80%E6%96%B0%E5%88%86%E6%9E%90%5C20120221_rmx%5Cannotation%5Crmx2_kegg%5Crmx2.htm#gene123) | 2 (0.51%) | ko04350 |
| 124 | [Ubiquitin mediated proteolysis](../../../../H:%5CP%E8%9B%8B%E7%99%BD%E8%B4%A8%E7%BB%84%E5%AD%A6%E6%96%87%E7%AB%A0%5C20120515%E5%89%AF%E6%80%A7%E8%85%BA%E5%92%8C%E7%B2%BE%E5%B7%A2%E8%9B%8B%E7%99%BD%E8%B4%A8%E7%BB%84%E6%9C%80%E6%96%B0%E5%88%86%E6%9E%90%5C20120221_rmx%5Cannotation%5Crmx2_kegg%5Crmx2.htm#gene124) | 2 (0.51%) | ko04120 |
| 125 | [N-Glycan biosynthesis](../../../../H:%5CP%E8%9B%8B%E7%99%BD%E8%B4%A8%E7%BB%84%E5%AD%A6%E6%96%87%E7%AB%A0%5C20120515%E5%89%AF%E6%80%A7%E8%85%BA%E5%92%8C%E7%B2%BE%E5%B7%A2%E8%9B%8B%E7%99%BD%E8%B4%A8%E7%BB%84%E6%9C%80%E6%96%B0%E5%88%86%E6%9E%90%5C20120221_rmx%5Cannotation%5Crmx2_kegg%5Crmx2.htm#gene125) | 1 (0.25%) | ko00510 |
| 126 | [Chagas disease (American trypanosomiasis)](../../../../H:%5CP%E8%9B%8B%E7%99%BD%E8%B4%A8%E7%BB%84%E5%AD%A6%E6%96%87%E7%AB%A0%5C20120515%E5%89%AF%E6%80%A7%E8%85%BA%E5%92%8C%E7%B2%BE%E5%B7%A2%E8%9B%8B%E7%99%BD%E8%B4%A8%E7%BB%84%E6%9C%80%E6%96%B0%E5%88%86%E6%9E%90%5C20120221_rmx%5Cannotation%5Crmx2_kegg%5Crmx2.htm#gene126) | 1 (0.25%) | ko05142 |
| 127 | [Basal transcription factors](../../../../H:%5CP%E8%9B%8B%E7%99%BD%E8%B4%A8%E7%BB%84%E5%AD%A6%E6%96%87%E7%AB%A0%5C20120515%E5%89%AF%E6%80%A7%E8%85%BA%E5%92%8C%E7%B2%BE%E5%B7%A2%E8%9B%8B%E7%99%BD%E8%B4%A8%E7%BB%84%E6%9C%80%E6%96%B0%E5%88%86%E6%9E%90%5C20120221_rmx%5Cannotation%5Crmx2_kegg%5Crmx2.htm#gene127) | 1 (0.25%) | ko03022 |
| 128 | [Vitamin digestion and absorption](../../../../H:%5CP%E8%9B%8B%E7%99%BD%E8%B4%A8%E7%BB%84%E5%AD%A6%E6%96%87%E7%AB%A0%5C20120515%E5%89%AF%E6%80%A7%E8%85%BA%E5%92%8C%E7%B2%BE%E5%B7%A2%E8%9B%8B%E7%99%BD%E8%B4%A8%E7%BB%84%E6%9C%80%E6%96%B0%E5%88%86%E6%9E%90%5C20120221_rmx%5Cannotation%5Crmx2_kegg%5Crmx2.htm#gene128) | 1 (0.25%) | ko04977 |
| 129 | [D-Glutamine and D-glutamate metabolism](../../../../H:%5CP%E8%9B%8B%E7%99%BD%E8%B4%A8%E7%BB%84%E5%AD%A6%E6%96%87%E7%AB%A0%5C20120515%E5%89%AF%E6%80%A7%E8%85%BA%E5%92%8C%E7%B2%BE%E5%B7%A2%E8%9B%8B%E7%99%BD%E8%B4%A8%E7%BB%84%E6%9C%80%E6%96%B0%E5%88%86%E6%9E%90%5C20120221_rmx%5Cannotation%5Crmx2_kegg%5Crmx2.htm#gene129) | 1 (0.25%) | ko00471 |
| 130 | [Lysine biosynthesis](../../../../H:%5CP%E8%9B%8B%E7%99%BD%E8%B4%A8%E7%BB%84%E5%AD%A6%E6%96%87%E7%AB%A0%5C20120515%E5%89%AF%E6%80%A7%E8%85%BA%E5%92%8C%E7%B2%BE%E5%B7%A2%E8%9B%8B%E7%99%BD%E8%B4%A8%E7%BB%84%E6%9C%80%E6%96%B0%E5%88%86%E6%9E%90%5C20120221_rmx%5Cannotation%5Crmx2_kegg%5Crmx2.htm#gene130) | 1 (0.25%) | ko00300 |
| 131 | [Autoimmune thyroid disease](../../../../H:%5CP%E8%9B%8B%E7%99%BD%E8%B4%A8%E7%BB%84%E5%AD%A6%E6%96%87%E7%AB%A0%5C20120515%E5%89%AF%E6%80%A7%E8%85%BA%E5%92%8C%E7%B2%BE%E5%B7%A2%E8%9B%8B%E7%99%BD%E8%B4%A8%E7%BB%84%E6%9C%80%E6%96%B0%E5%88%86%E6%9E%90%5C20120221_rmx%5Cannotation%5Crmx2_kegg%5Crmx2.htm#gene131) | 1 (0.25%) | ko05320 |
| 132 | [Caffeine metabolism](../../../../H:%5CP%E8%9B%8B%E7%99%BD%E8%B4%A8%E7%BB%84%E5%AD%A6%E6%96%87%E7%AB%A0%5C20120515%E5%89%AF%E6%80%A7%E8%85%BA%E5%92%8C%E7%B2%BE%E5%B7%A2%E8%9B%8B%E7%99%BD%E8%B4%A8%E7%BB%84%E6%9C%80%E6%96%B0%E5%88%86%E6%9E%90%5C20120221_rmx%5Cannotation%5Crmx2_kegg%5Crmx2.htm#gene132) | 1 (0.25%) | ko00232 |
| 133 | [Base excision repair](../../../../H:%5CP%E8%9B%8B%E7%99%BD%E8%B4%A8%E7%BB%84%E5%AD%A6%E6%96%87%E7%AB%A0%5C20120515%E5%89%AF%E6%80%A7%E8%85%BA%E5%92%8C%E7%B2%BE%E5%B7%A2%E8%9B%8B%E7%99%BD%E8%B4%A8%E7%BB%84%E6%9C%80%E6%96%B0%E5%88%86%E6%9E%90%5C20120221_rmx%5Cannotation%5Crmx2_kegg%5Crmx2.htm#gene133) | 1 (0.25%) | ko03410 |
| 134 | [Thyroid cancer](../../../../H:%5CP%E8%9B%8B%E7%99%BD%E8%B4%A8%E7%BB%84%E5%AD%A6%E6%96%87%E7%AB%A0%5C20120515%E5%89%AF%E6%80%A7%E8%85%BA%E5%92%8C%E7%B2%BE%E5%B7%A2%E8%9B%8B%E7%99%BD%E8%B4%A8%E7%BB%84%E6%9C%80%E6%96%B0%E5%88%86%E6%9E%90%5C20120221_rmx%5Cannotation%5Crmx2_kegg%5Crmx2.htm#gene134) | 1 (0.25%) | ko05216 |
| 135 | [mTOR signaling pathway](../../../../H:%5CP%E8%9B%8B%E7%99%BD%E8%B4%A8%E7%BB%84%E5%AD%A6%E6%96%87%E7%AB%A0%5C20120515%E5%89%AF%E6%80%A7%E8%85%BA%E5%92%8C%E7%B2%BE%E5%B7%A2%E8%9B%8B%E7%99%BD%E8%B4%A8%E7%BB%84%E6%9C%80%E6%96%B0%E5%88%86%E6%9E%90%5C20120221_rmx%5Cannotation%5Crmx2_kegg%5Crmx2.htm#gene135) | 1 (0.25%) | ko04150 |
| 136 | [Colorectal cancer](../../../../H:%5CP%E8%9B%8B%E7%99%BD%E8%B4%A8%E7%BB%84%E5%AD%A6%E6%96%87%E7%AB%A0%5C20120515%E5%89%AF%E6%80%A7%E8%85%BA%E5%92%8C%E7%B2%BE%E5%B7%A2%E8%9B%8B%E7%99%BD%E8%B4%A8%E7%BB%84%E6%9C%80%E6%96%B0%E5%88%86%E6%9E%90%5C20120221_rmx%5Cannotation%5Crmx2_kegg%5Crmx2.htm#gene136) | 1 (0.25%) | ko05210 |
| 137 | [Carbohydrate digestion and absorption](../../../../H:%5CP%E8%9B%8B%E7%99%BD%E8%B4%A8%E7%BB%84%E5%AD%A6%E6%96%87%E7%AB%A0%5C20120515%E5%89%AF%E6%80%A7%E8%85%BA%E5%92%8C%E7%B2%BE%E5%B7%A2%E8%9B%8B%E7%99%BD%E8%B4%A8%E7%BB%84%E6%9C%80%E6%96%B0%E5%88%86%E6%9E%90%5C20120221_rmx%5Cannotation%5Crmx2_kegg%5Crmx2.htm#gene137) | 1 (0.25%) | ko04973 |
| 138 | [Terpenoid backbone biosynthesis](../../../../H:%5CP%E8%9B%8B%E7%99%BD%E8%B4%A8%E7%BB%84%E5%AD%A6%E6%96%87%E7%AB%A0%5C20120515%E5%89%AF%E6%80%A7%E8%85%BA%E5%92%8C%E7%B2%BE%E5%B7%A2%E8%9B%8B%E7%99%BD%E8%B4%A8%E7%BB%84%E6%9C%80%E6%96%B0%E5%88%86%E6%9E%90%5C20120221_rmx%5Cannotation%5Crmx2_kegg%5Crmx2.htm#gene138) | 1 (0.25%) | ko00900 |
| 139 | [Ether lipid metabolism](../../../../H:%5CP%E8%9B%8B%E7%99%BD%E8%B4%A8%E7%BB%84%E5%AD%A6%E6%96%87%E7%AB%A0%5C20120515%E5%89%AF%E6%80%A7%E8%85%BA%E5%92%8C%E7%B2%BE%E5%B7%A2%E8%9B%8B%E7%99%BD%E8%B4%A8%E7%BB%84%E6%9C%80%E6%96%B0%E5%88%86%E6%9E%90%5C20120221_rmx%5Cannotation%5Crmx2_kegg%5Crmx2.htm#gene139) | 1 (0.25%) | ko00565 |
| 140 | [Jak-STAT signaling pathway](../../../../H:%5CP%E8%9B%8B%E7%99%BD%E8%B4%A8%E7%BB%84%E5%AD%A6%E6%96%87%E7%AB%A0%5C20120515%E5%89%AF%E6%80%A7%E8%85%BA%E5%92%8C%E7%B2%BE%E5%B7%A2%E8%9B%8B%E7%99%BD%E8%B4%A8%E7%BB%84%E6%9C%80%E6%96%B0%E5%88%86%E6%9E%90%5C20120221_rmx%5Cannotation%5Crmx2_kegg%5Crmx2.htm#gene140) | 1 (0.25%) | ko04630 |
| 141 | [Epithelial cell signaling in Helicobacter pylori infection](../../../../H:%5CP%E8%9B%8B%E7%99%BD%E8%B4%A8%E7%BB%84%E5%AD%A6%E6%96%87%E7%AB%A0%5C20120515%E5%89%AF%E6%80%A7%E8%85%BA%E5%92%8C%E7%B2%BE%E5%B7%A2%E8%9B%8B%E7%99%BD%E8%B4%A8%E7%BB%84%E6%9C%80%E6%96%B0%E5%88%86%E6%9E%90%5C20120221_rmx%5Cannotation%5Crmx2_kegg%5Crmx2.htm#gene141) | 1 (0.25%) | ko05120 |
| 142 | [Progesterone-mediated oocyte maturation](../../../../H:%5CP%E8%9B%8B%E7%99%BD%E8%B4%A8%E7%BB%84%E5%AD%A6%E6%96%87%E7%AB%A0%5C20120515%E5%89%AF%E6%80%A7%E8%85%BA%E5%92%8C%E7%B2%BE%E5%B7%A2%E8%9B%8B%E7%99%BD%E8%B4%A8%E7%BB%84%E6%9C%80%E6%96%B0%E5%88%86%E6%9E%90%5C20120221_rmx%5Cannotation%5Crmx2_kegg%5Crmx2.htm#gene142) | 1 (0.25%) | ko04914 |
| 143 | [Long-term depression](../../../../H:%5CP%E8%9B%8B%E7%99%BD%E8%B4%A8%E7%BB%84%E5%AD%A6%E6%96%87%E7%AB%A0%5C20120515%E5%89%AF%E6%80%A7%E8%85%BA%E5%92%8C%E7%B2%BE%E5%B7%A2%E8%9B%8B%E7%99%BD%E8%B4%A8%E7%BB%84%E6%9C%80%E6%96%B0%E5%88%86%E6%9E%90%5C20120221_rmx%5Cannotation%5Crmx2_kegg%5Crmx2.htm#gene143) | 1 (0.25%) | ko04730 |
| 144 | [T cell receptor signaling pathway](../../../../H:%5CP%E8%9B%8B%E7%99%BD%E8%B4%A8%E7%BB%84%E5%AD%A6%E6%96%87%E7%AB%A0%5C20120515%E5%89%AF%E6%80%A7%E8%85%BA%E5%92%8C%E7%B2%BE%E5%B7%A2%E8%9B%8B%E7%99%BD%E8%B4%A8%E7%BB%84%E6%9C%80%E6%96%B0%E5%88%86%E6%9E%90%5C20120221_rmx%5Cannotation%5Crmx2_kegg%5Crmx2.htm#gene144) | 1 (0.25%) | ko04660 |
| 145 | [Type I diabetes mellitus](../../../../H:%5CP%E8%9B%8B%E7%99%BD%E8%B4%A8%E7%BB%84%E5%AD%A6%E6%96%87%E7%AB%A0%5C20120515%E5%89%AF%E6%80%A7%E8%85%BA%E5%92%8C%E7%B2%BE%E5%B7%A2%E8%9B%8B%E7%99%BD%E8%B4%A8%E7%BB%84%E6%9C%80%E6%96%B0%E5%88%86%E6%9E%90%5C20120221_rmx%5Cannotation%5Crmx2_kegg%5Crmx2.htm#gene145) | 1 (0.25%) | ko04940 |
| 146 | [One carbon pool by folate](../../../../H:%5CP%E8%9B%8B%E7%99%BD%E8%B4%A8%E7%BB%84%E5%AD%A6%E6%96%87%E7%AB%A0%5C20120515%E5%89%AF%E6%80%A7%E8%85%BA%E5%92%8C%E7%B2%BE%E5%B7%A2%E8%9B%8B%E7%99%BD%E8%B4%A8%E7%BB%84%E6%9C%80%E6%96%B0%E5%88%86%E6%9E%90%5C20120221_rmx%5Cannotation%5Crmx2_kegg%5Crmx2.htm#gene146) | 1 (0.25%) | ko00670 |
| 147 | [Valine, leucine and isoleucine biosynthesis](../../../../H:%5CP%E8%9B%8B%E7%99%BD%E8%B4%A8%E7%BB%84%E5%AD%A6%E6%96%87%E7%AB%A0%5C20120515%E5%89%AF%E6%80%A7%E8%85%BA%E5%92%8C%E7%B2%BE%E5%B7%A2%E8%9B%8B%E7%99%BD%E8%B4%A8%E7%BB%84%E6%9C%80%E6%96%B0%E5%88%86%E6%9E%90%5C20120221_rmx%5Cannotation%5Crmx2_kegg%5Crmx2.htm#gene147) | 1 (0.25%) | ko00290 |
| 148 | [ECM-receptor interaction](../../../../H:%5CP%E8%9B%8B%E7%99%BD%E8%B4%A8%E7%BB%84%E5%AD%A6%E6%96%87%E7%AB%A0%5C20120515%E5%89%AF%E6%80%A7%E8%85%BA%E5%92%8C%E7%B2%BE%E5%B7%A2%E8%9B%8B%E7%99%BD%E8%B4%A8%E7%BB%84%E6%9C%80%E6%96%B0%E5%88%86%E6%9E%90%5C20120221_rmx%5Cannotation%5Crmx2_kegg%5Crmx2.htm#gene148) | 1 (0.25%) | ko04512 |
| 149 | [Pancreatic cancer](../../../../H:%5CP%E8%9B%8B%E7%99%BD%E8%B4%A8%E7%BB%84%E5%AD%A6%E6%96%87%E7%AB%A0%5C20120515%E5%89%AF%E6%80%A7%E8%85%BA%E5%92%8C%E7%B2%BE%E5%B7%A2%E8%9B%8B%E7%99%BD%E8%B4%A8%E7%BB%84%E6%9C%80%E6%96%B0%E5%88%86%E6%9E%90%5C20120221_rmx%5Cannotation%5Crmx2_kegg%5Crmx2.htm#gene149) | 1 (0.25%) | ko05212 |
| 150 | [Cytokine-cytokine receptor interaction](../../../../H:%5CP%E8%9B%8B%E7%99%BD%E8%B4%A8%E7%BB%84%E5%AD%A6%E6%96%87%E7%AB%A0%5C20120515%E5%89%AF%E6%80%A7%E8%85%BA%E5%92%8C%E7%B2%BE%E5%B7%A2%E8%9B%8B%E7%99%BD%E8%B4%A8%E7%BB%84%E6%9C%80%E6%96%B0%E5%88%86%E6%9E%90%5C20120221_rmx%5Cannotation%5Crmx2_kegg%5Crmx2.htm#gene150) | 1 (0.25%) | ko04060 |
